# Supplementary material for: Bromotryptamine and Bromotyramine Derivatives from the Tropical Southwestern Pacific Sponge Narrabeena nigra
Source: Mar Drugs. 2019 May 30;17(6):319. doi: 10.3390/md17060319 (PMC6627171; doi:10.3390/md17060319)
Supplement: Supplementary file 1 [file marinedrugs-17-00319-s001.pdf]

Bromotryptamine and bromotyramine derivatives from the Tropical Southwestern Pacific sponge *Narrabeena nigra*.

Maria Miguel Gordo,[1] Kevin Calabro,[1] Sandra Gegunde,[2] Laurence Jennings,[1] Amparo Alfonso,[2] Grégory Genta-Jouve,[3] Jean Vacelet,[4] Luis M. Botana,[2] and Olivier P. Thomas*,[1]

[1] Marine Biodiscovery, School of Chemistry and Ryan Institute, National University of Ireland Galway (NUI Galway), University Road, H91 TK33 Galway, Ireland; m.miguelgordo1@nuigalway.ie (M.M.G.), kevin.calabro@nuigalway.ie (K.C.), laurence.jennings@nuigalway.ie (L.J.),

[2] Departamento de Farmacología, Facultad de Veterinaria, Universidade de Santiago de Compostela, 27002 Lugo, Spain; sandra.gegunde@rai.usc.es (S.G.); amparo.alfonso@usc.es (A.A.); luis.botana@usc.es (L.M.B.)

[3] Laboratoire de Chimie-Toxicologie Analytique et Cellulaire (C-TAC) UMR CNRS 8638 COMETE, Université Paris-Descartes, 4, avenue de l'Observatoire, 75006 Paris, France; gregory.genta-jouve@parisdescartes.fr (G.G.J.)

[4] Aix Marseille Université, CNRS, IRD, IMBE UMR 7263, Avignon Université, Institut Méditerranéen de Biodiversité et d'Ecologie marine et continentale, Station Marine d'Endoume, Chemin de la Batterie des Lions, 13007 Marseille, France; jean.vacelet@imbe.fr (J.V.)

* Correspondence: olivier.thomas@nuigalway.ie; Tel.: +353-9149-3563 (O.P.T.)

S1. *Narrabeena nigra* specimen collected at Alofi Island. 5
S2. ^{1}H NMR data, δ_H in ppm, mult. (J in Hz), in MeOH-d_4 for bromotryptamines 5
S3. ^{1}H NMR data, δ_H in ppm, mult. (J in Hz), in MeOH-d_4 for bromotyramines 5
S4. ESI(+)-HRMS analysis of **1** and crop of the molecular ion. 6
S5. ^{1}H NMR spectrum of **1** (600 MHz, MeOH-d_4). 6
S6. COSY NMR spectrum of **1** (600 MHz, MeOH-d_4). 7
S7. HSQC NMR spectrum of **1** (600 MHz, MeOH-d_4). 7
S8. HMBC NMR spectrum of **1** (600 MHz, MeOH-d_4). 8
S9. ESI(+)-HRMS analysis of **2** and crop of the molecular ion. 9
S10. ^{1}H NMR spectrum of **2** (600 MHz, MeOH-d_4). 9
S11. ^{13}C NMR spectrum of **2** (150 MHz, MeOH-d_4). 10
S12. COSY NMR spectrum of **2** (600 MHz, MeOH-d_4). 10
S13. HSQC NMR spectrum of **2** (600 MHz, MeOH-d_4). 11
S14. HMBC NMR spectrum of **2** (600 MHz, MeOH-d_4). 11
S15. ESI(+)-HRMS analysis of **3** and crop of the molecular ion. 12
S16. ^{1}H NMR spectrum of **3** (500 MHz, MeOH-d_4). 12
S17. ^{13}C NMR spectrum of **3** (125 MHz, MeOH-d_4). 13
S18. HSQC NMR spectrum of **3** (500 MHz, MeOH-d_4). 13
S19. HMBC NMR spectrum of **3** (500 MHz, MeOH-d_4). 14
S20. ESI(+)-HRMS analysis of **4** and crop of the molecular ion. 15
S21. ^{1}H NMR spectrum of **4** (600 MHz, MeOH-d_4). 15
S22. ^{13}C NMR spectrum of **4** (150 MHz, MeOH-d_4). 16
S23. COSY NMR spectrum of **4** (600 MHz, MeOH-d_4). 16
S24. HSQC NMR spectrum of **4** (600 MHz, MeOH-d_4). 17
S25. HMBC NMR spectrum of **4** (600 MHz, MeOH-d_4). 17
S26. ECD spectrum of compound **4** in CH_3CN at 0.1 mg/mL 18
S27. ESI(+)-HRMS analysis of **5** and crop of the molecular ion. 19
S28. ^{1}H NMR spectrum of **5** (500 MHz, MeOH-d_4). 19
S29. ^{13}C NMR spectrum of **5** (125 MHz, MeOH-d_4). 20
S30. COSY NMR spectrum of **5** (500 MHz, MeOH-d_4). 20
S31. HSQC NMR spectrum of **5** (500 MHz, MeOH-d_4). 21
S32. HMBC NMR spectrum of **5** (500 MHz, MeOH-d_4). 21
S33. ECD spectrum of compound **5** in CH_3CN at 0.2 mg/mL 22
S34. ESI(+)-HRMS analysis of **6** and crop of the molecular ion. 23

S35. ^{1}H NMR spectrum of **6** (500 MHz, MeOH-d_4). 23
S36. ^{13}C NMR spectrum of **6** (125 MHz, MeOH-d_4). 24
S37. COSY NMR spectrum of **6** (500 MHz, MeOH-d_4). 24
S38. HSQC NMR spectrum of **6** (500 MHz, MeOH-d_4). 25
S39. HMBC NMR spectrum of **6** (500 MHz, MeOH-d_4). 25
S40. ESI(+)-HRMS analysis of **7** and crop of the molecular ion. 26
S41. ^{1}H NMR spectrum of **7** (500 MHz, MeOH-d_4). 26
S42. ^{13}C NMR spectrum of **7** (125 MHz, MeOH-d_4). 27
S43. HSQC NMR spectrum of **7** (500 MHz, MeOH-d_4). 27
S44. HMBC NMR spectrum of **7** (500 MHz, MeOH-d_4). 28
S45. ESI(+)-HRMS analysis of **8** and crop of the molecular ion. 29
S46. ^{1}H NMR spectrum of **8** (500 MHz, MeOH-d_4). 29
S47. ^{13}C NMR spectrum of **8** (125 MHz, MeOH-d_4). 30
S48. COSY NMR spectrum of **8** (500 MHz, MeOH-d_4). 30
S49. HSQC NMR spectrum of **8** (500 MHz, MeOH-d_4). 31
S50. HMBC NMR spectrum of **8** (500 MHz, MeOH-d_4). 31
S51. ESI(+)-HRMS analysis of **9** 32
S52. ^{1}H NMR spectrum of **9** (500 MHz, MeOH-d_4). 32
S53. ^{13}C NMR spectrum of **9** (125 MHz, MeOH-d_4). 33
S54. COSY NMR spectrum of **9** (500 MHz, MeOH-d_4). 33
S55. HSQC NMR spectrum of **9** (500 MHz, MeOH-d_4). 34
S56. HSQC NMR spectrum of **9** (500 MHz, MeOH-d_4). 34
S57. ESI(+)-HRMS analysis of **10** 35
S58. ^{1}H NMR spectrum of **10** (500 MHz, MeOH-d_4). 35
S59. ESI(+)-HRMS analysis of **11** 36
S60. ^{1}H NMR spectrum of **11** (500 MHz, MeOH-d_4). 36
S61. ESI(+)-HRMS analysis of **12** 37
S62. ^{1}H NMR spectrum of **12** (500 MHz, MeOH-d_4). 37
S63. ESI(+)-HRMS analysis of **13** 38
S64. ^{1}H NMR spectrum of **13** (500 MHz, MeOH-d_4). 38
S65. ESI(+)-HRMS analysis of **14** 39
S66. ^{1}H NMR spectrum of **14** (500 MHz, MeOH-d_4). 39
S67. ESI(+)-HRMS analysis of **15** 40
S68. ^{1}H NMR spectrum of **15** (500 MHz, MeOH-d_4). 40
S69. ESI(+)-HRMS analysis of **16** 41
S70. ^{1}H NMR spectrum of **16** (500 MHz, MeOH-d_4). 41

S71. ^{13}C NMR spectrum of **16** (125 MHz, MeOH-d_4). 42
S72. COSY NMR spectrum of **16** (500 MHz, MeOH-d_4)........ 42
S73. HSQC NMR spectrum of **16** (500 MHz, MeOH-d_4). 43
S74. HMBC NMR spectrum of **16** (500 MHz, MeOH-d_4). 43
S75. ESI(+)-HRMS analysis of **17**........ 44
S76. ^{1}H NMR spectrum of **17** (500 MHz, MeOH-d_4). 44
S77. ESI(+)-HRMS analysis of **18**........ 45
S78. ^{1}H NMR spectrum of **18** (500 MHz, MeOH-d_4). 45
S79. Cell viability of brominated alkaloids over microglia BV2 cell line. 46
S80. Cell viability of brominated alkaloids over neuroblastoma SH-SY5Y cell line. 47
S81. Cosine values of all newly identified compounds in orange in the MetWork software........ 48
S82. Comparison between the experimental and calculated MS/MS spectrum of the minor compound below. 48

Biological material.

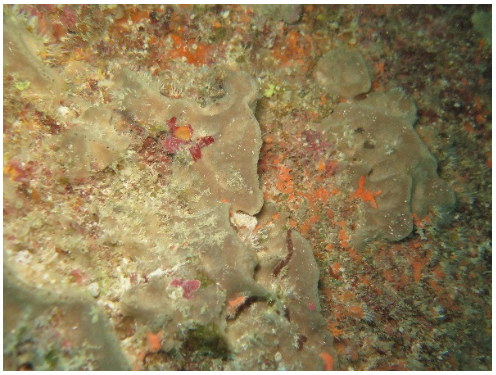

S1. *Narrabeena nigra* specimen collected at Alofi Island.

Irregularly lamellar or thickly encrusting sponge, beige gray in life, surface becoming dark grey in alcohol. Skeleton of uncored, laminated fibers, 15-100 µm in diameter, arranged in an irregular reticulation with meshes 150-500 µm in size. Primary fibers 50-100 µm in diameter, with a small central pith, ending free on 350-500 µm at the surface. Secondary fibers 15-50 µm in diameter, without pith.

S2. ^{1}H NMR data, δ_H in ppm, mult. (J in Hz), in MeOH-d_4 for bromotryptamines

No.	9[b]	10[a]	11[a]	12[a]	13[a]	14[a]	15[a]
**2**	7.26, s	7.26, s	7.24, s	7.20, s	7.19, s		
**4**	7.93, s	7.95, s	7.93, s	7.49, d (8.5)	7.49, d (8.5)	7.63, d (8.5)	8.15, d (8.5)
**5**	-	-	-	7.17, dd (8.5, 1.5)	7.17, dd (8.5, 1.5)	6.72, dd (8.5, 1.5)	7.55, dd (8.5, 1.5)
**6**	-	-	-	-			
**7**	7.70, s	7.74, s	7.73, s	7.54, d (1.5)	7.54, d (1.5)	6.98, d (1.5)	7.80, d (1.5)
**7a**	-	-	-	-	-	-	-
**8**	3.14, t (7.5)	3.10, t (7.5)	3.07, t (7.5)	3.13, t (7.5)	3.10, t (7.5)	[c]	6.35, d (7.5)
**9**	3.39, t (7.5)	3.29, t (7.5)	3.21, t (7.5)	3.30[c]	3.22, t (7.5)	3.34, t (5.5)	7.99, d (7.5)
**11**	2.93, s			2.70, s			

[a] 500MHz [b] 600MHz [c] Overlap with solvent signal

S3. ^{1}H NMR data, δ_H in ppm, mult. (J in Hz), in MeOH-d_4 for bromotyramines

No.	16[a]	17[a]	18[a]
**2**	7.53, s	7.48, d (1.5)	7.51, d (1.5)
**3**	-	-	-
**4**	-	-	-
**5**	-	7.01, d (8.5)	6.98, d (8.5)
**6**	7.53, s	7.22, dd (8.5, 1.5)	7.26, dd (8.5, 1.5)
**7**	2.90, t (7.5)	2.87, d (7.5)	3.96, d (12.0)
**8**	3.16, t (7.5)	3.13, d (7.5)	3.13, t (12.0)
**10**			3.29, s
**O-C$\underline{H}_3$**	3.84, s	3.86, s	3.85, s

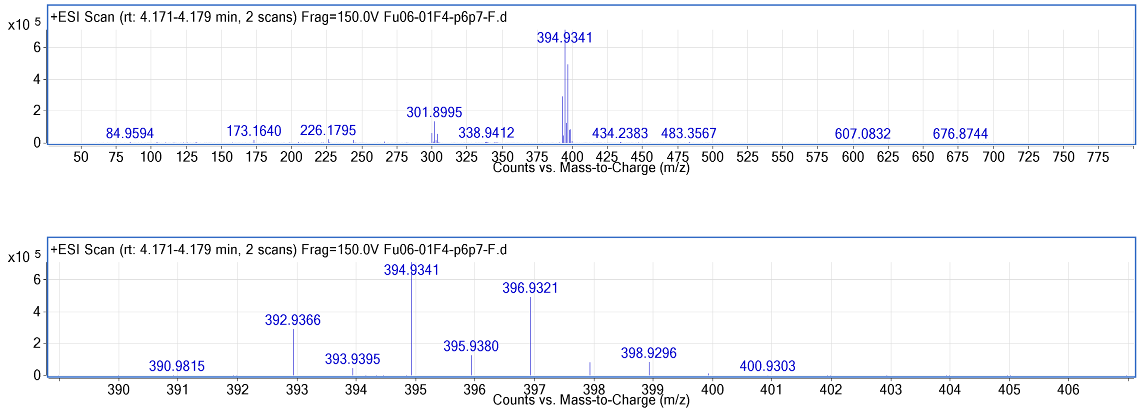

S4. ESI(+)-HRMS analysis of **1** and crop of the molecular ion.

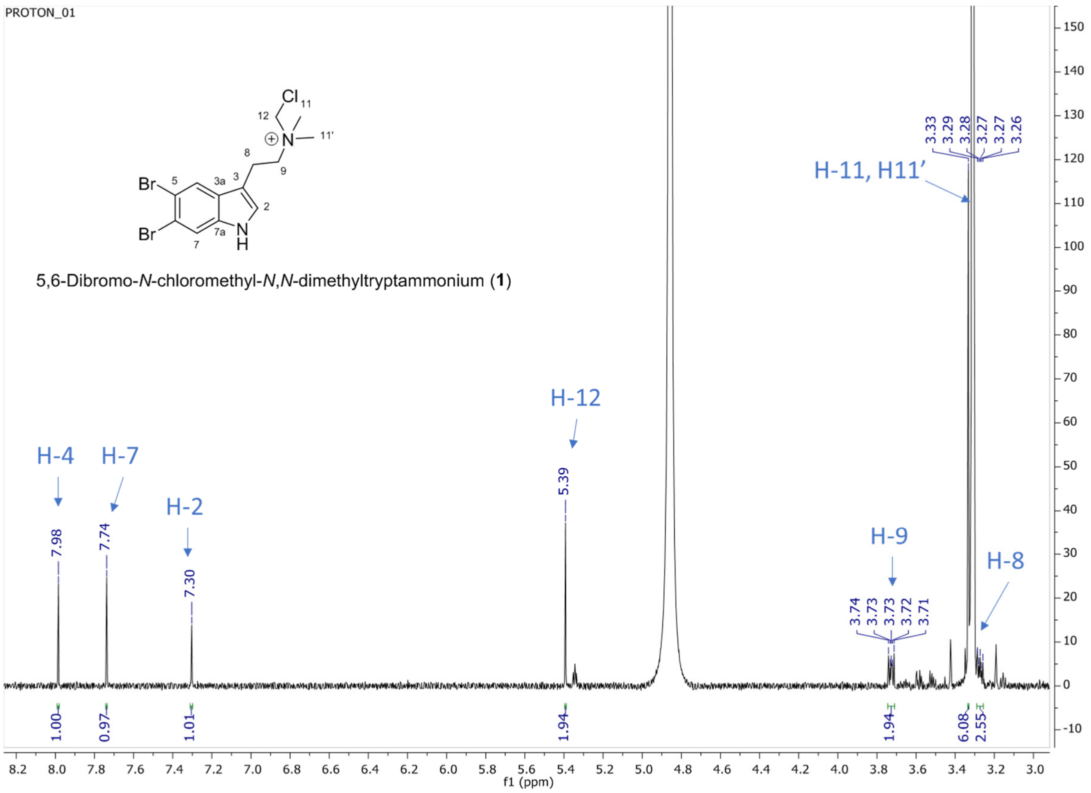

S5. ^{1}H NMR spectrum of **1** (600 MHz, MeOH-d_4).

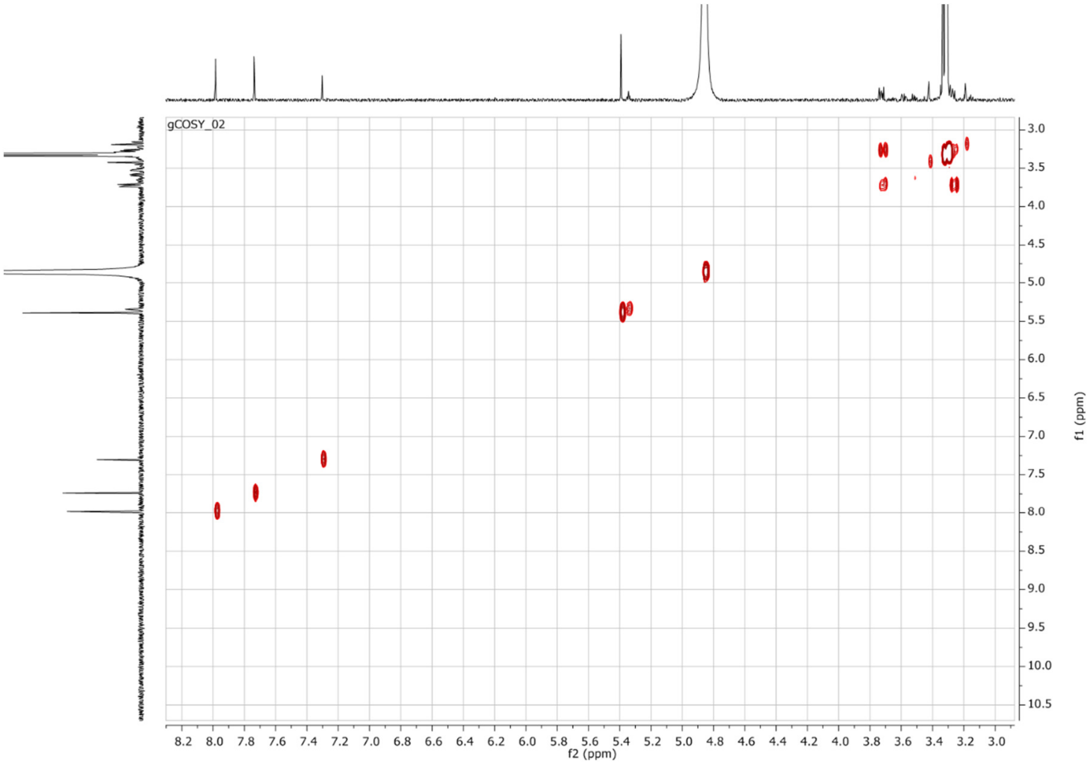

S6. COSY NMR spectrum of **1** (600 MHz, MeOH-d_4).

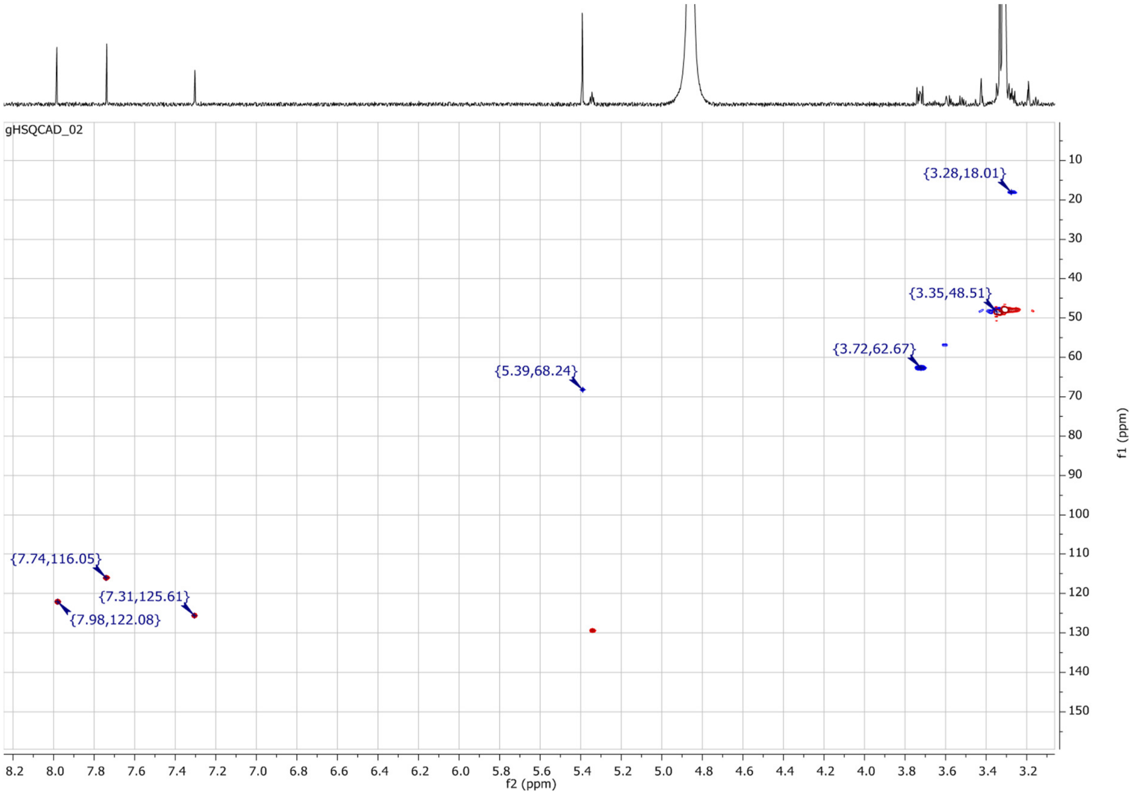

S7. HSQC NMR spectrum of **1** (600 MHz, MeOH-d_4).

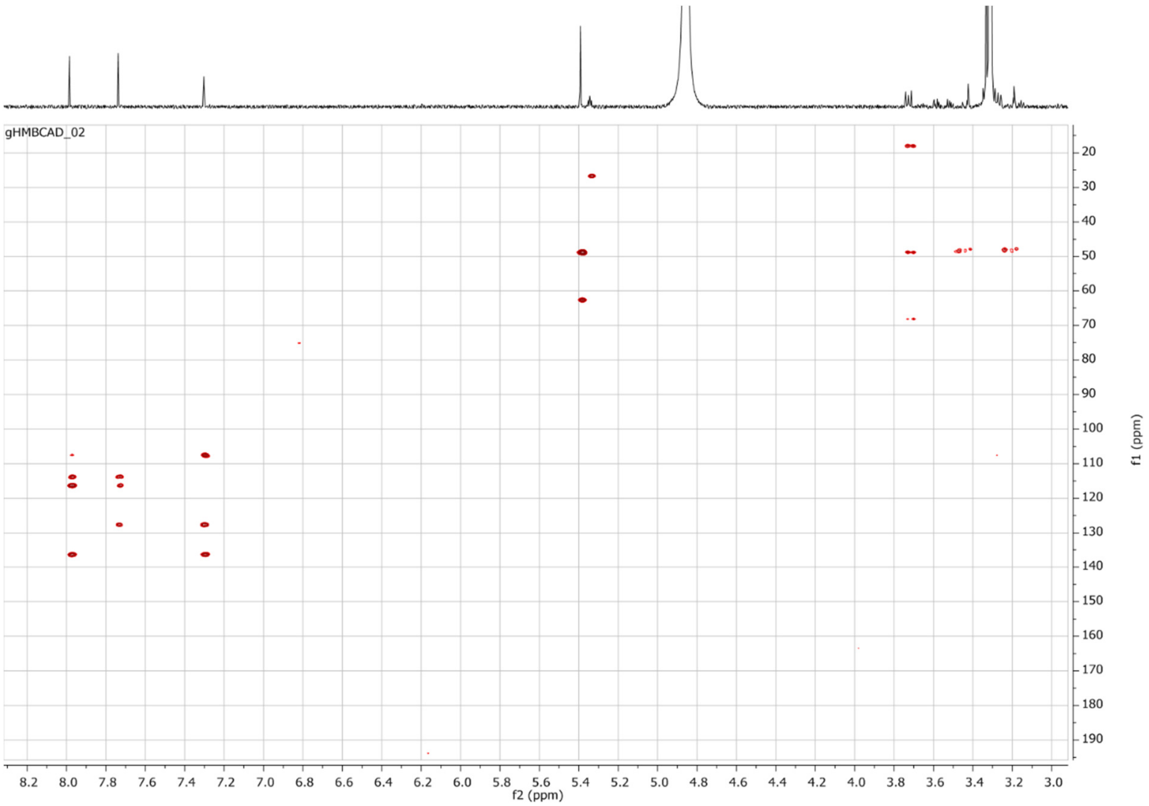

S8. HMBC NMR spectrum of **1** (600 MHz, MeOH-d_4).

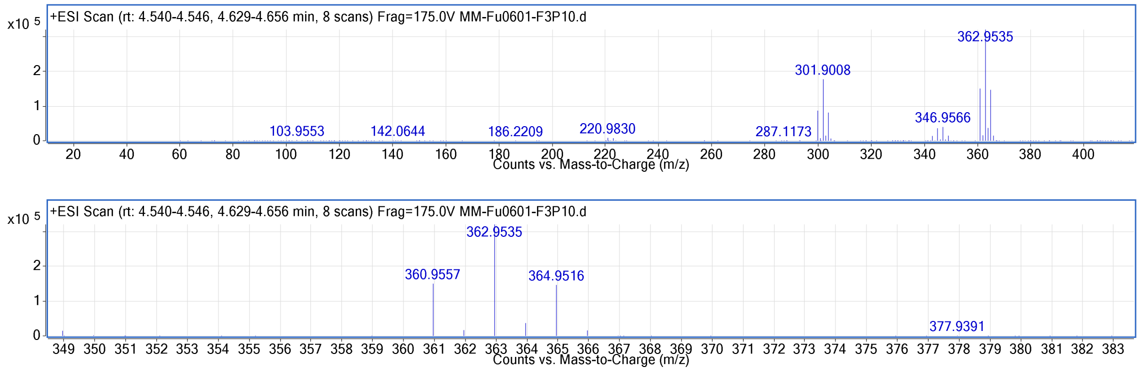

S9. ESI(+)-HRMS analysis of **2** and crop of the molecular ion.

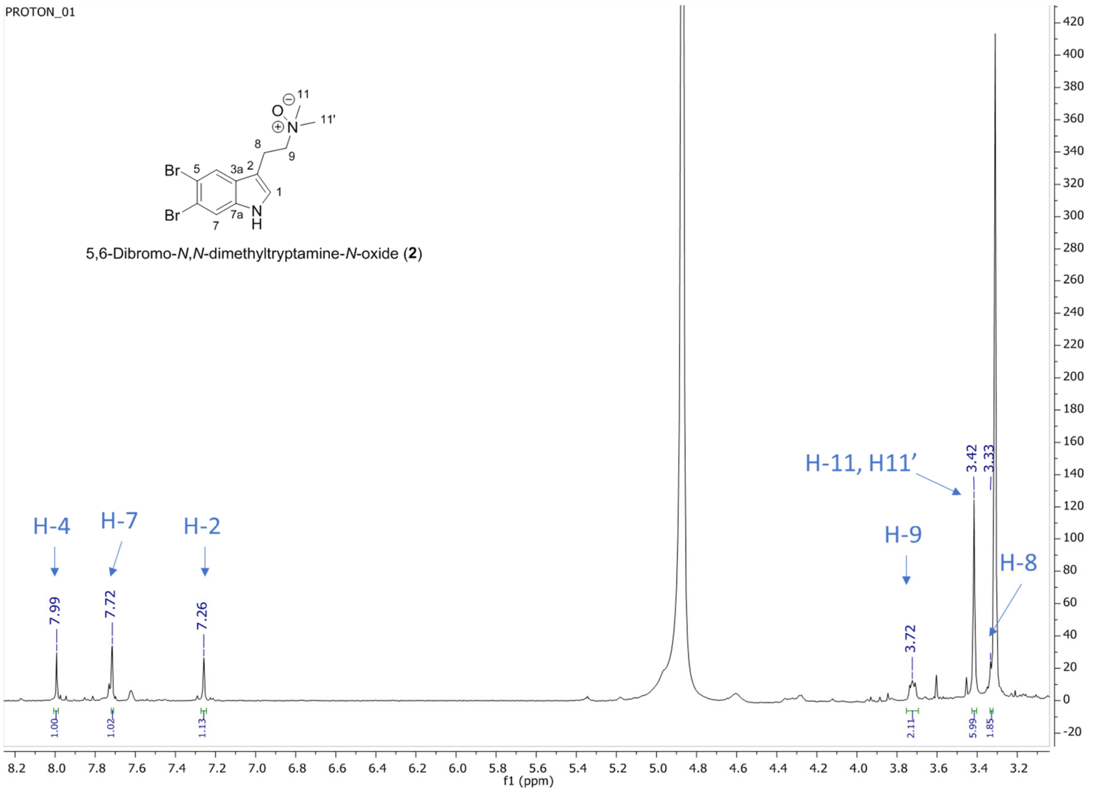

S10. ^{1}H NMR spectrum of **2** (600 MHz, MeOH-*d*$_4$).

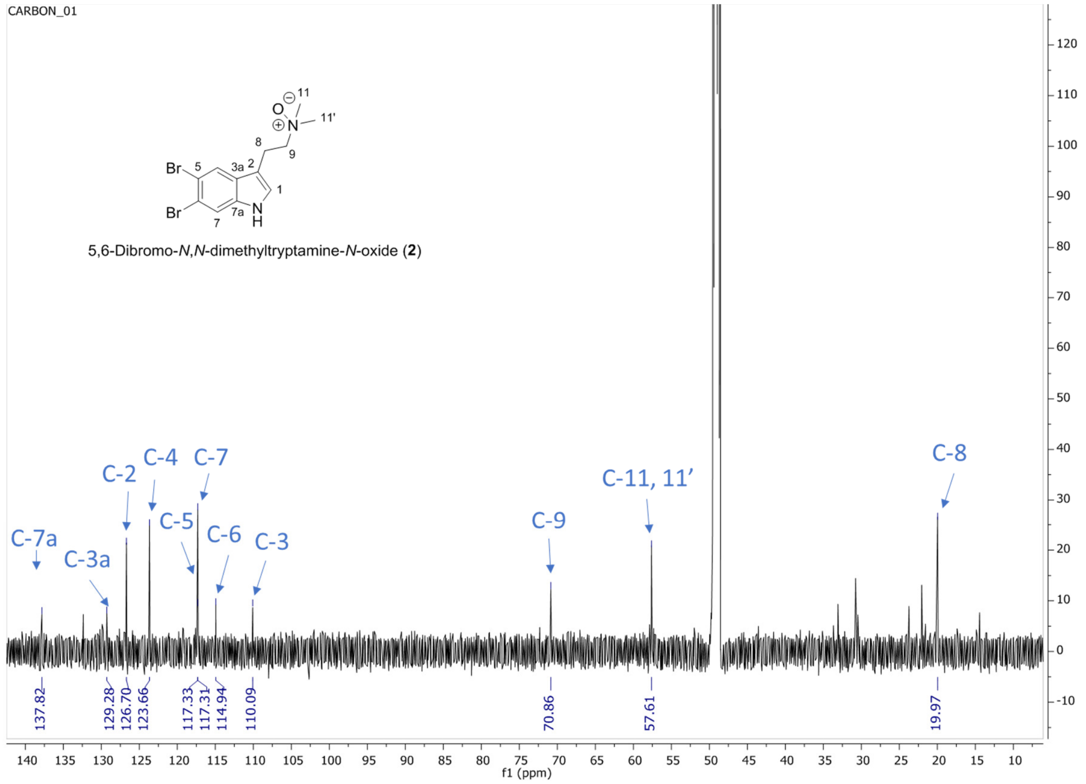

S11. ^{13}C NMR spectrum of **2** (150 MHz, MeOH-d_4).

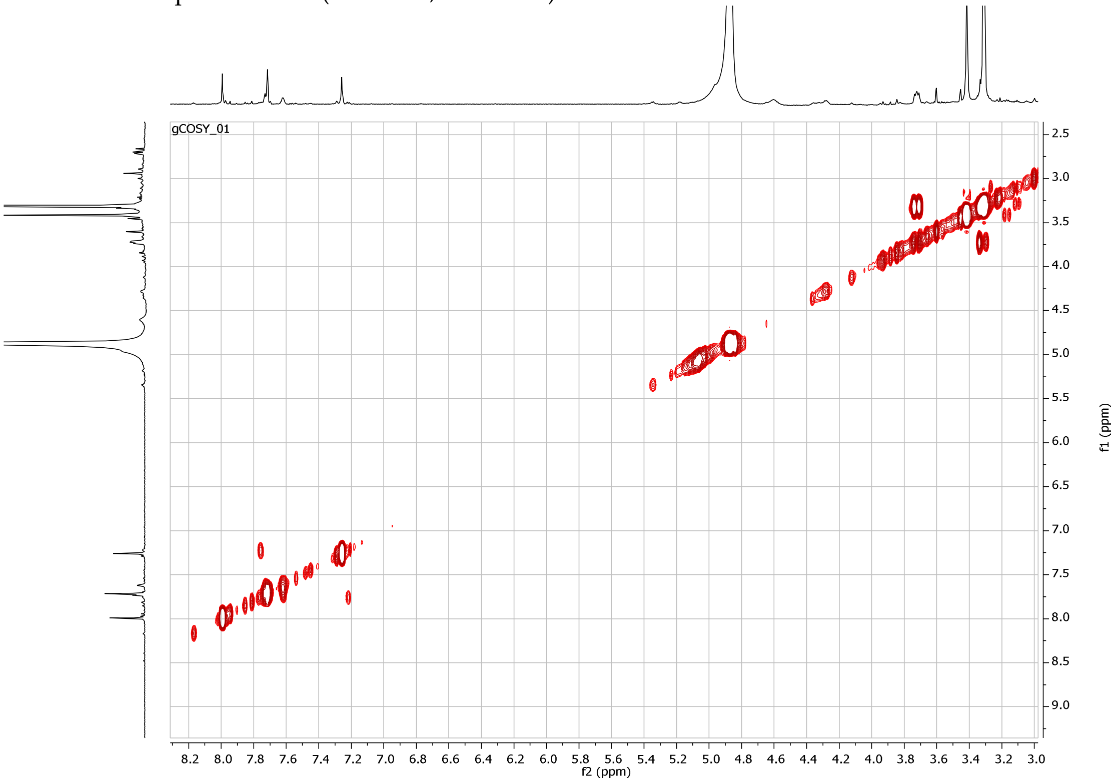

S12. COSY NMR spectrum of **2** (600 MHz, MeOH-d_4).

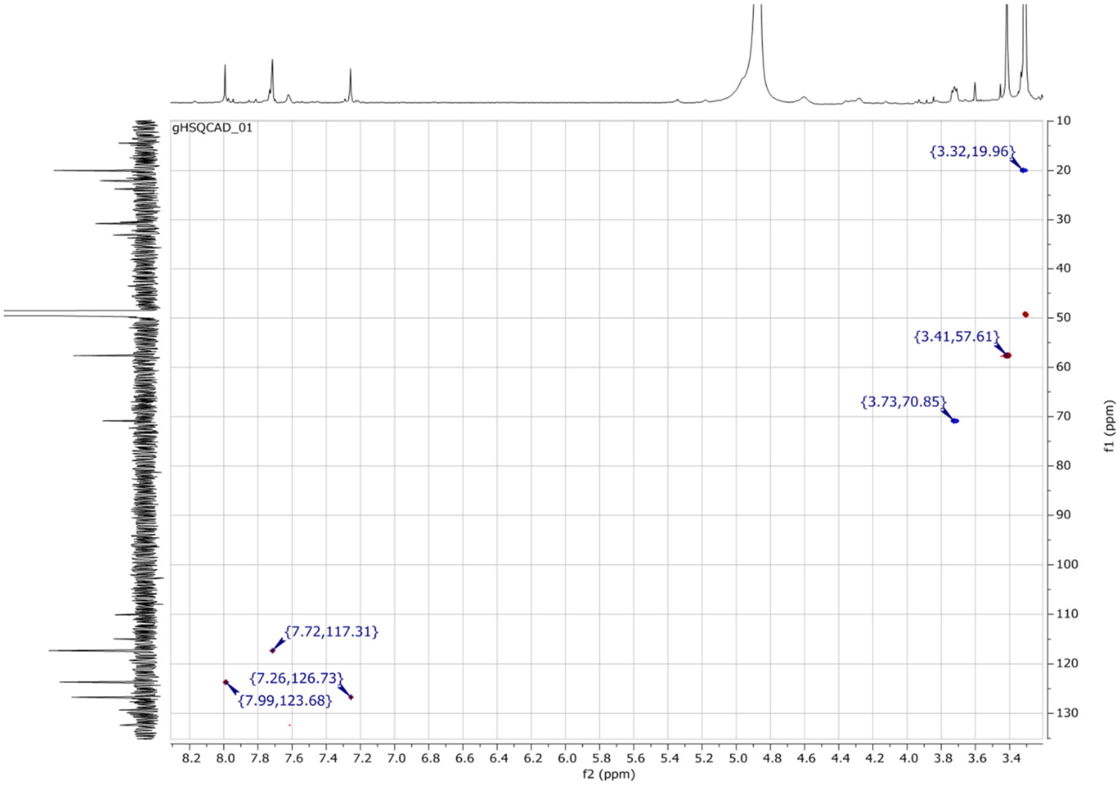

S13. HSQC NMR spectrum of **2** (600 MHz, MeOH-d_4).

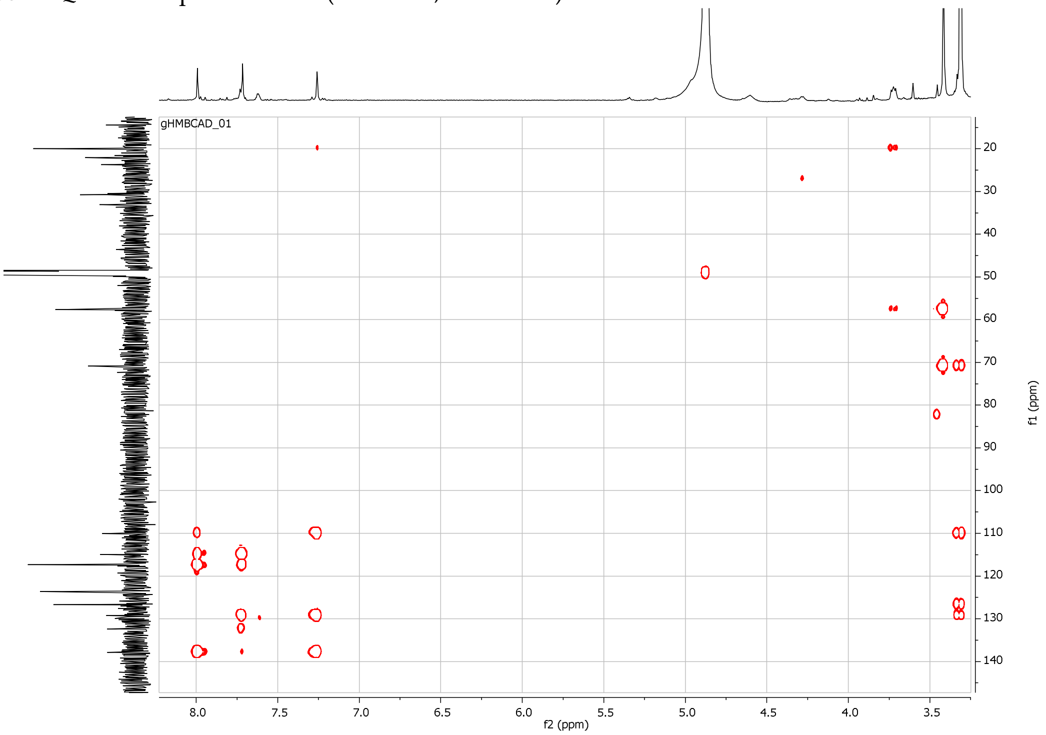

S14. HMBC NMR spectrum of **2** (600 MHz, MeOH-d_4).

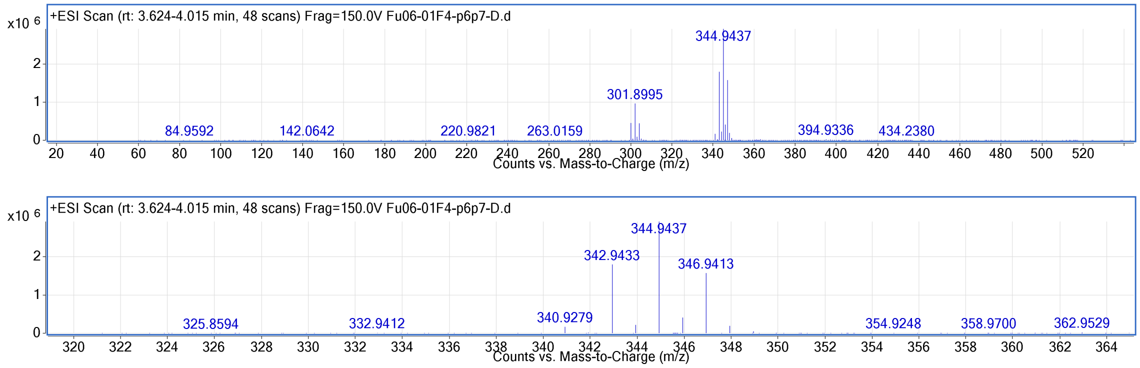

S15. ESI(+)-HRMS analysis of **3** and crop of the molecular ion.

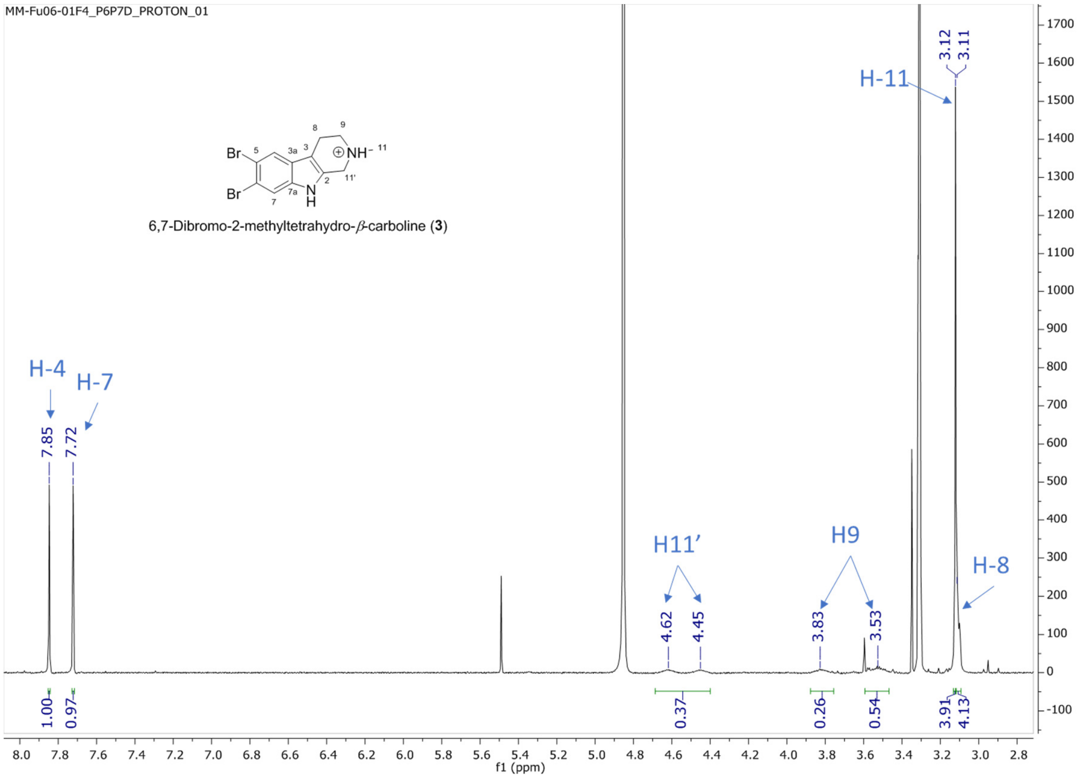

S16. ^{1}H NMR spectrum of **3** (500 MHz, MeOH-d_4).

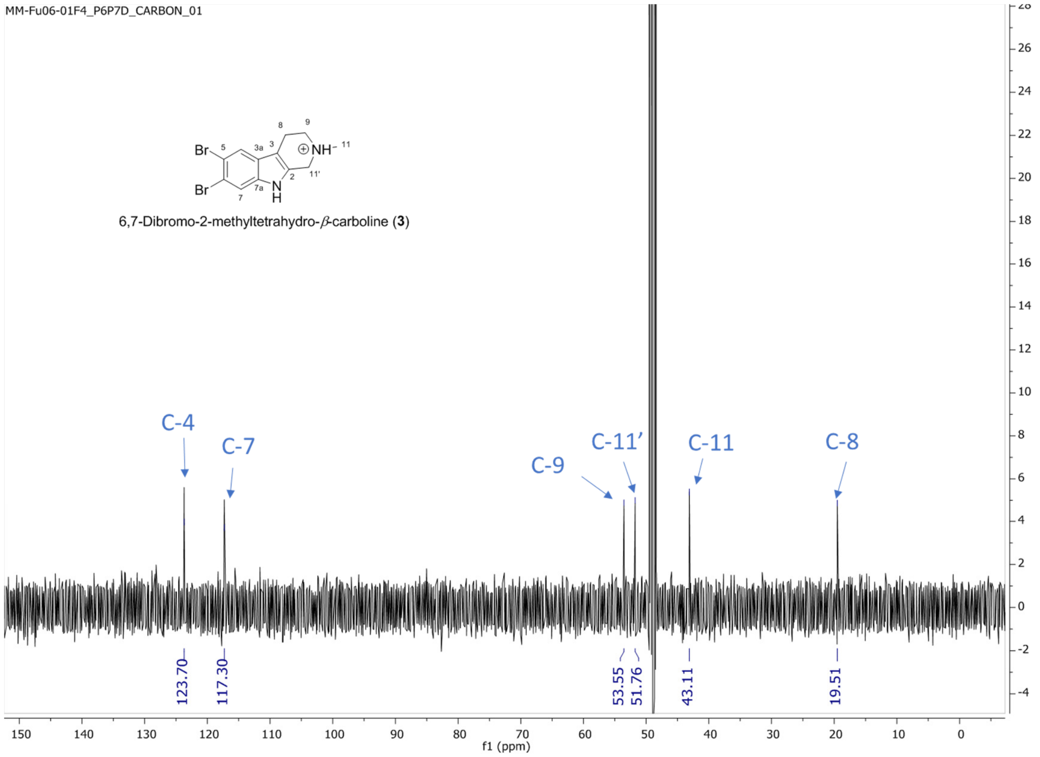

S17. ^{13}C NMR spectrum of **3** (125 MHz, MeOH-d_4).

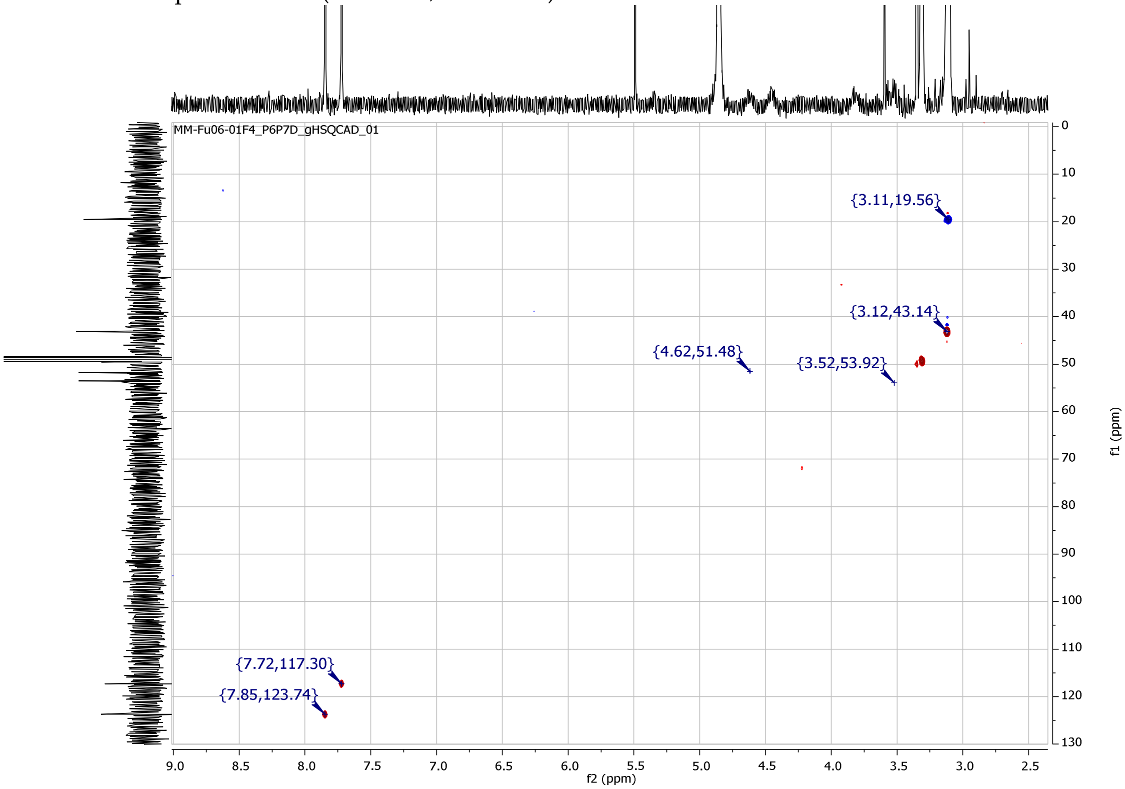

S18. HSQC NMR spectrum of **3** (500 MHz, MeOH-d_4).

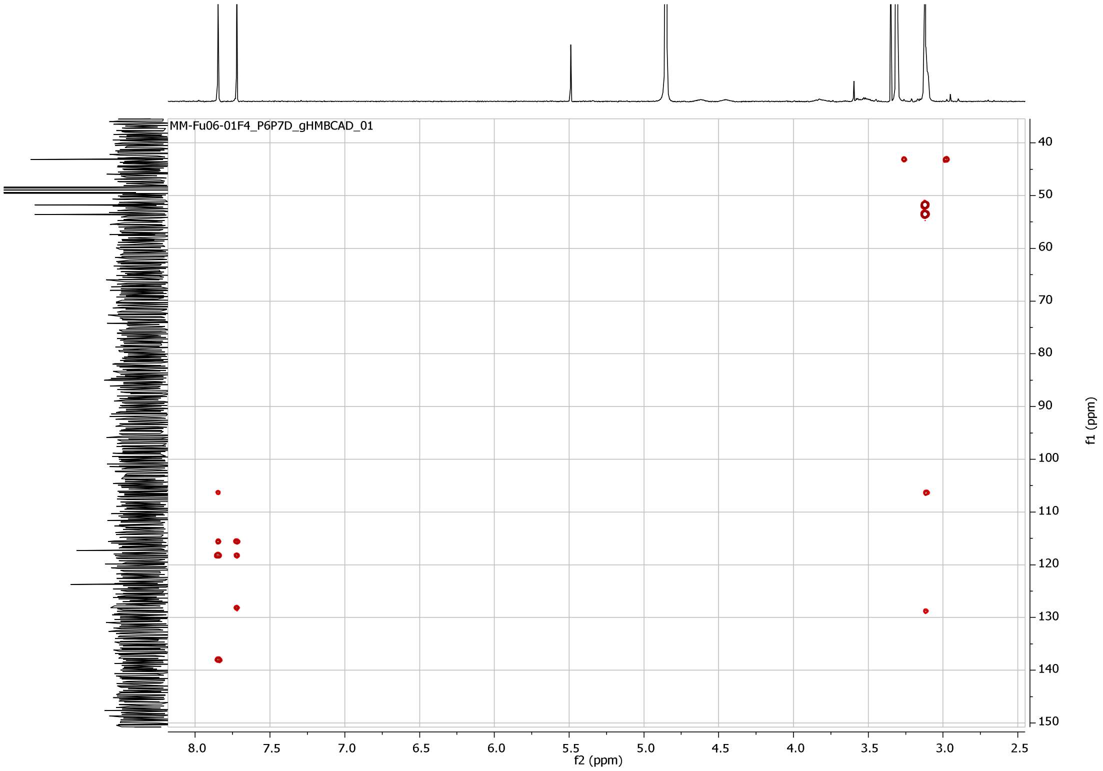

S19. HMBC NMR spectrum of **3** (500 MHz, MeOH-d_4).

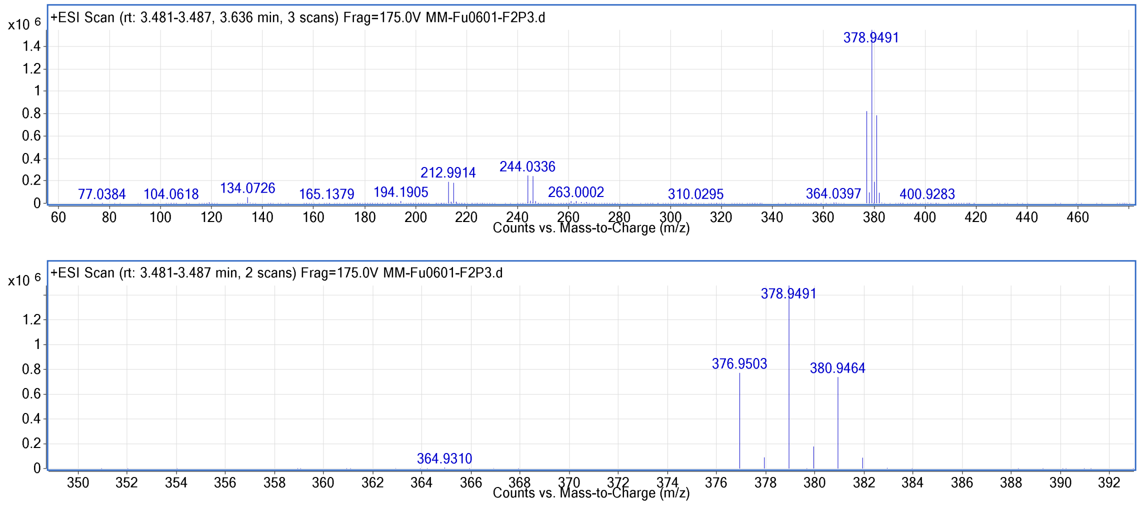

S20. ESI(+)-HRMS analysis of **4** and crop of the molecular ion.

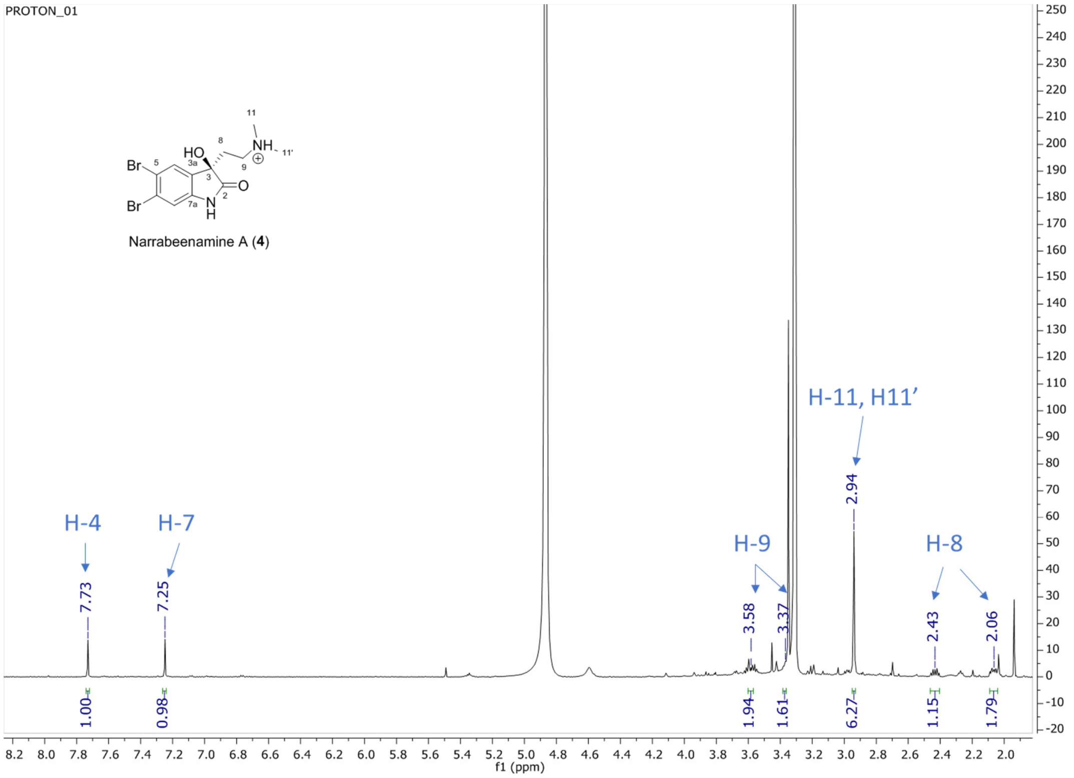

S21. ^{1}H NMR spectrum of **4** (600 MHz, MeOH-d_4).

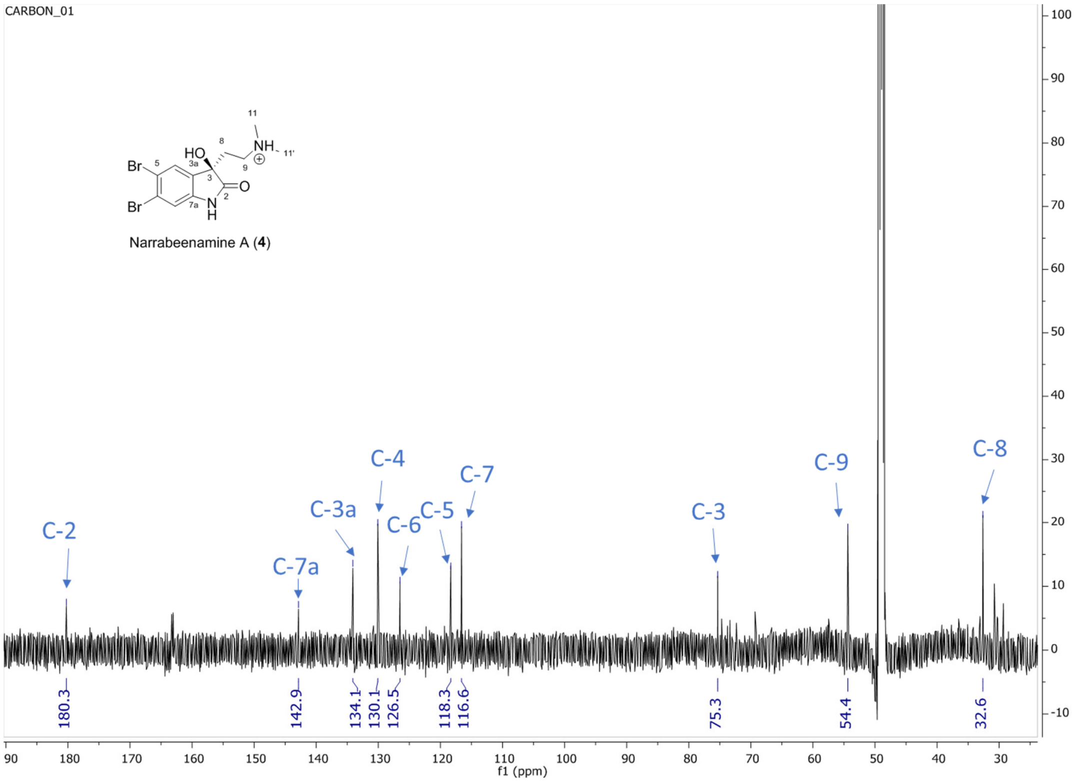

S22. ^{13}C NMR spectrum of **4** (150 MHz, MeOH-d_4).

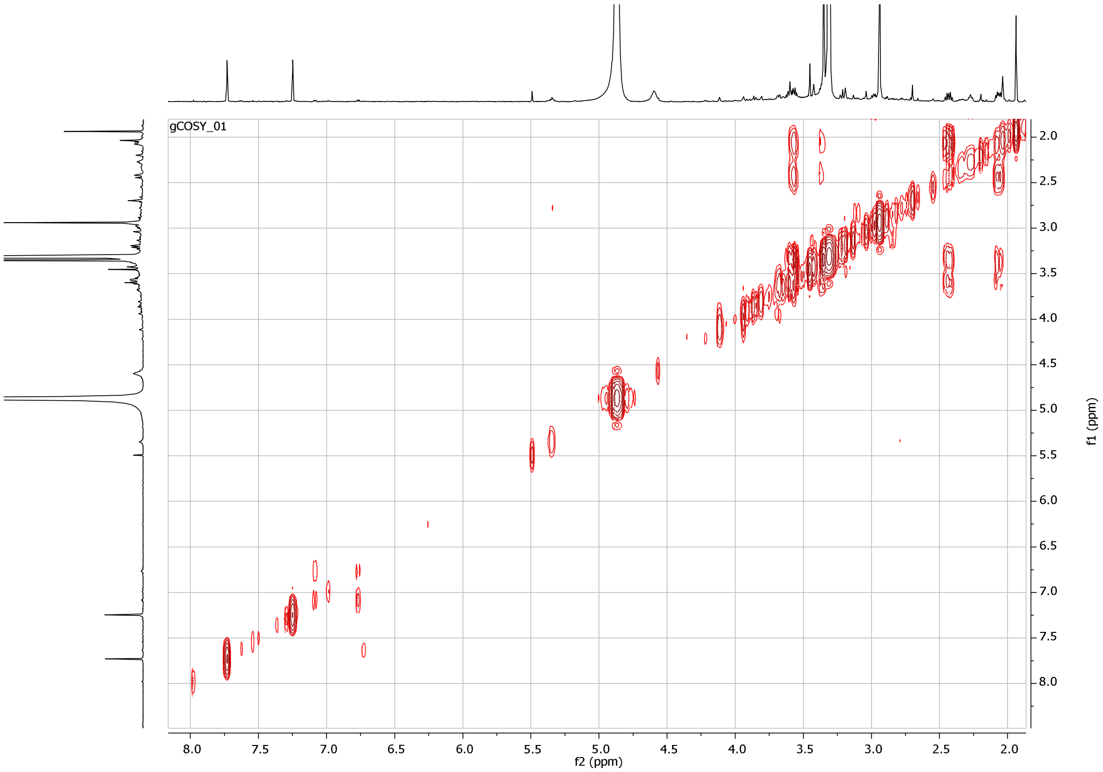

S23. COSY NMR spectrum of **4** (600 MHz, MeOH-d_4).

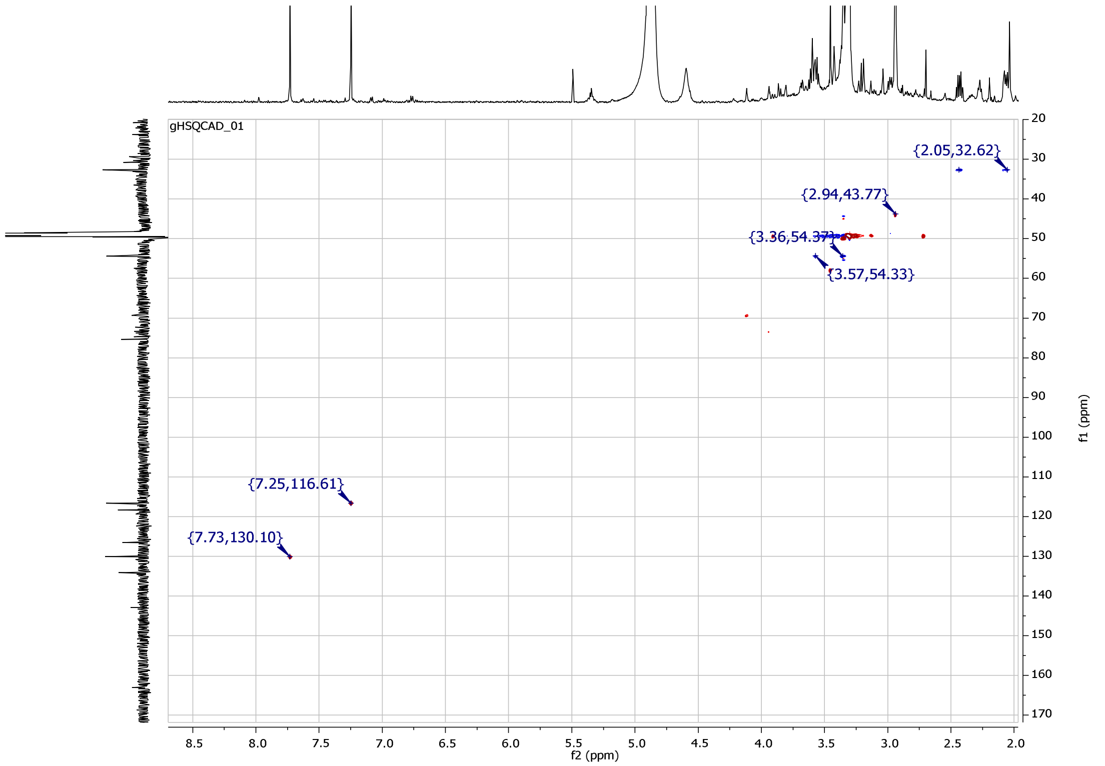

S24. HSQC NMR spectrum of **4** (600 MHz, MeOH-d_4).

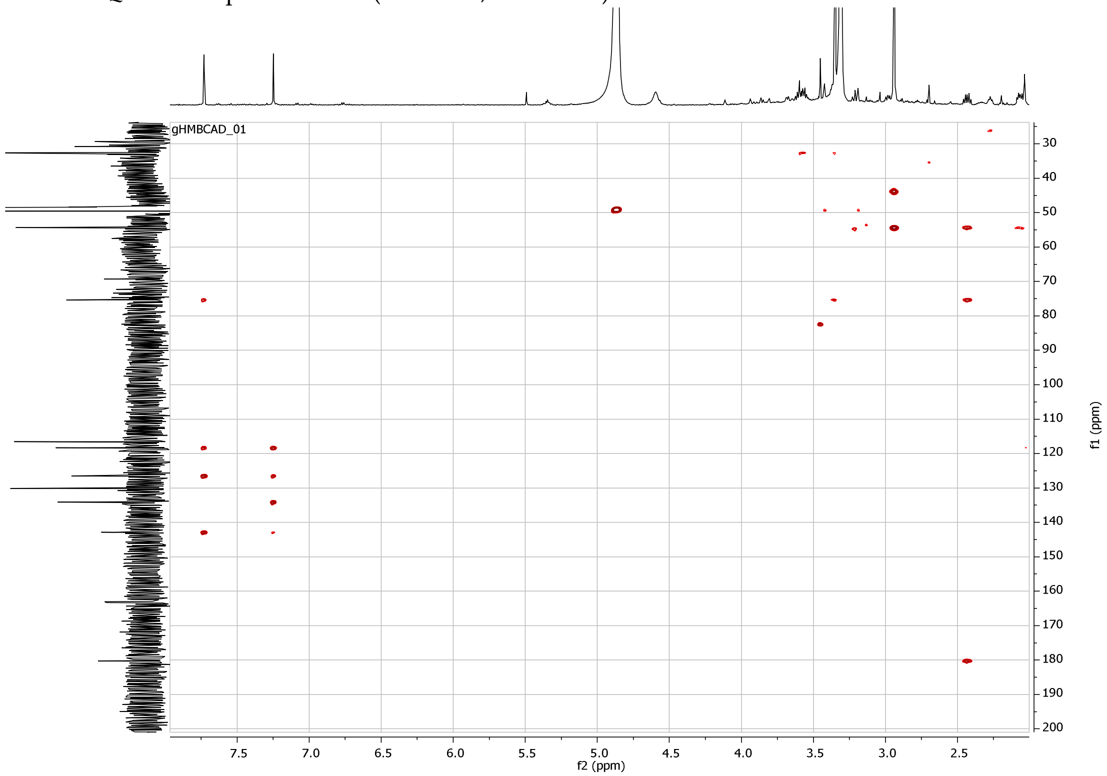

S25. HMBC NMR spectrum of **4** (600 MHz, MeOH-d_4).

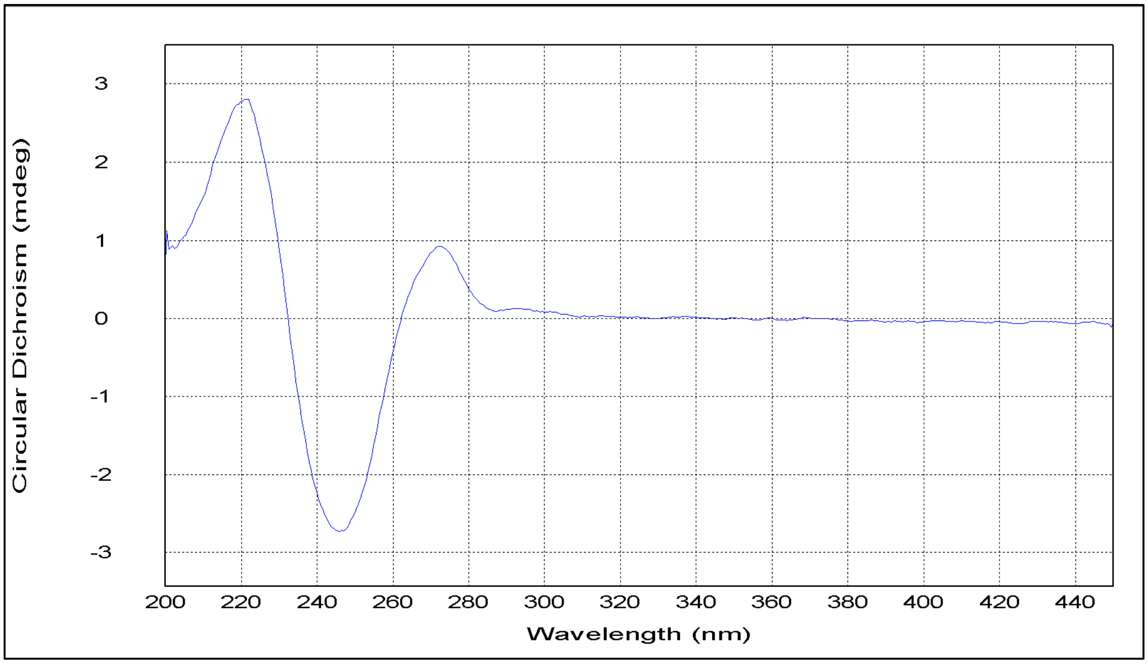

S26. ECD spectrum of compound **4** in CH_3CN at 0.1 mg/mL

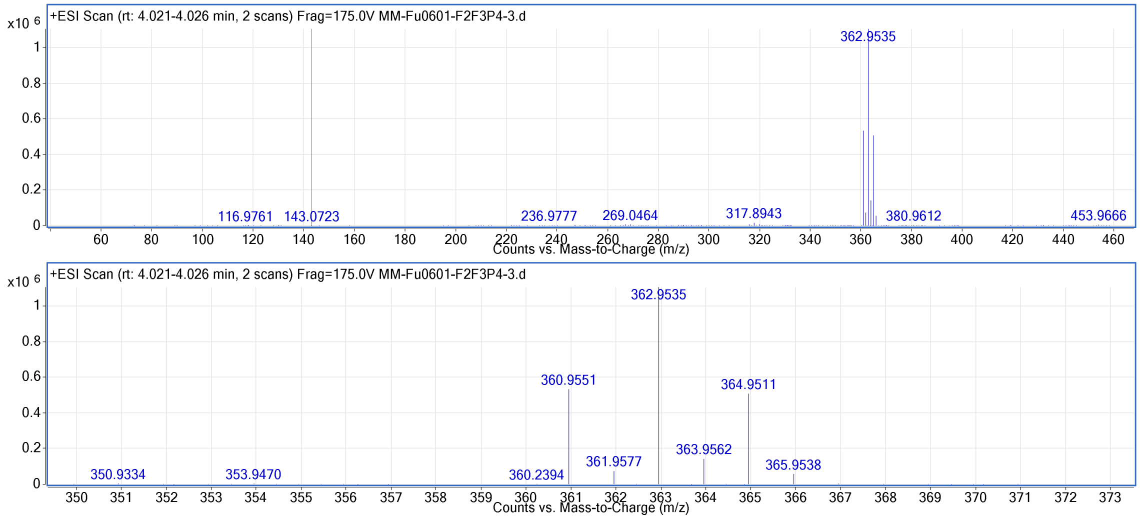

S27. ESI(+)-HRMS analysis of **5** and crop of the molecular ion.

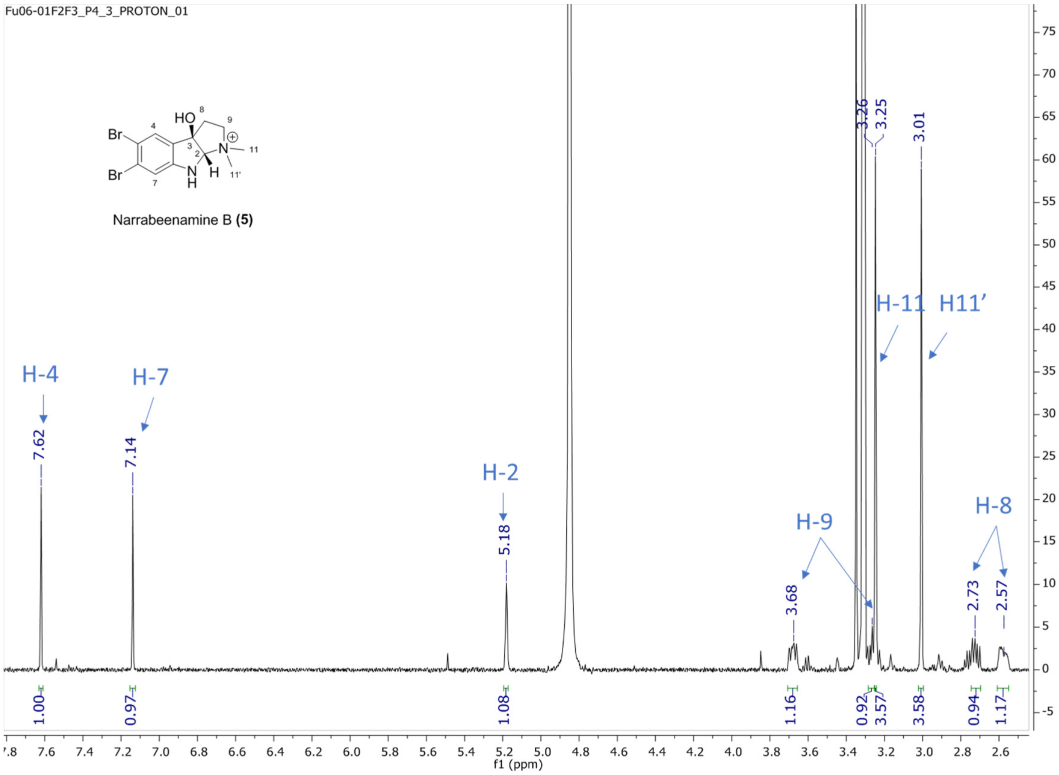

S28. ^{1}H NMR spectrum of **5** (500 MHz, MeOH-d_4).

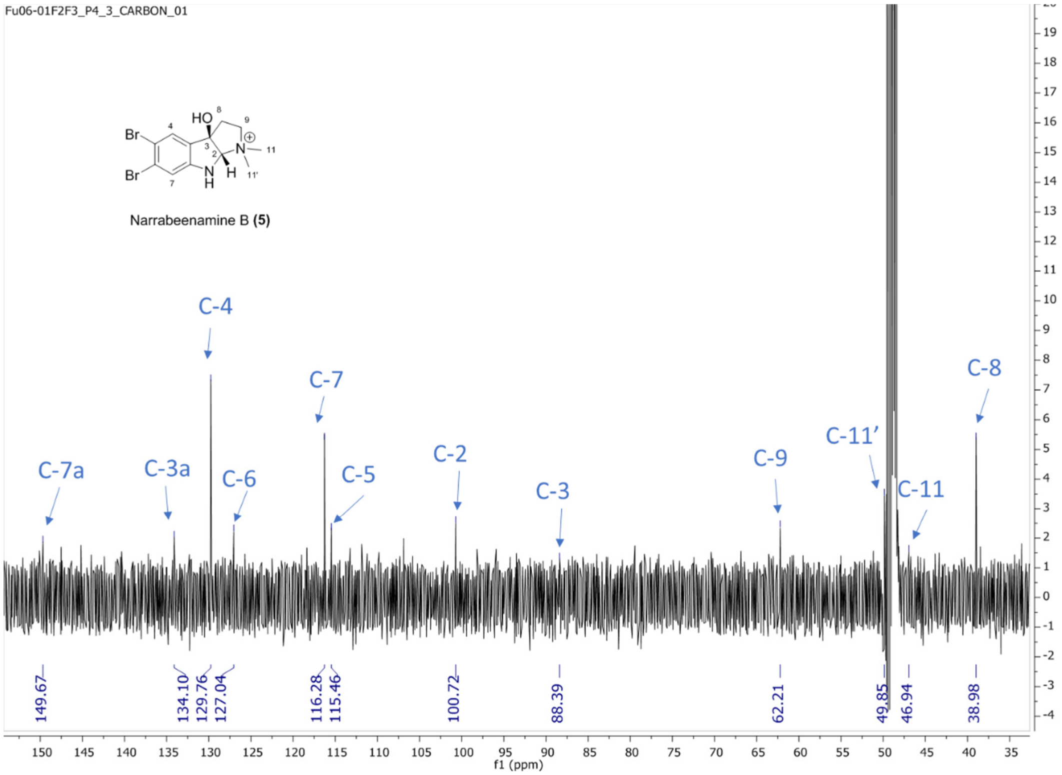

S29. ^{13}C NMR spectrum of **5** (125 MHz, MeOH-d_4).

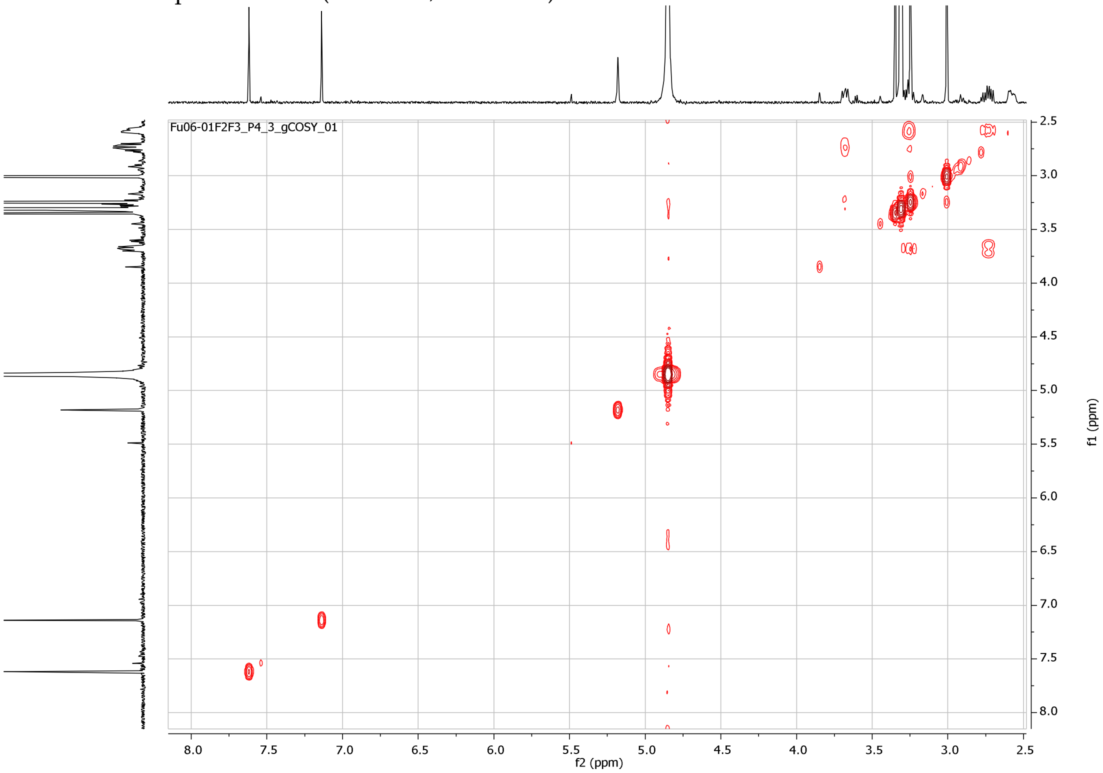

S30. COSY NMR spectrum of **5** (500 MHz, MeOH-d_4).

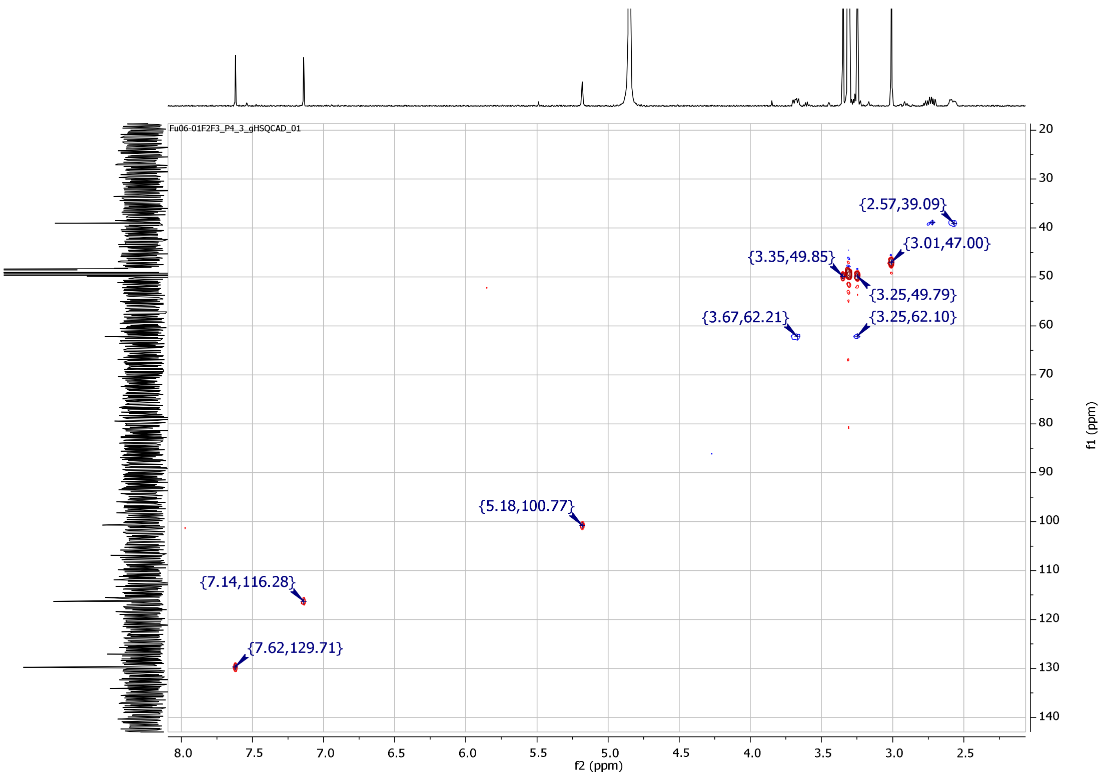

S31. HSQC NMR spectrum of **5** (500 MHz, MeOH-d_4).

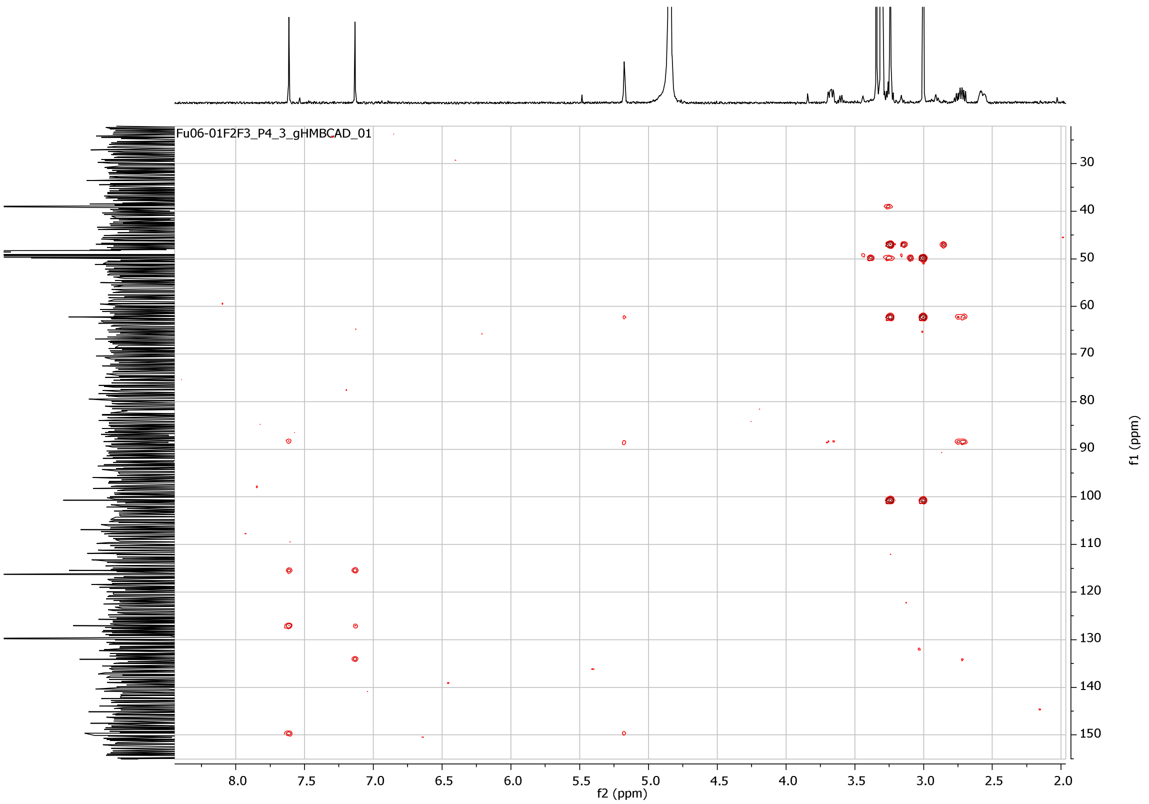

S32. HMBC NMR spectrum of **5** (500 MHz, MeOH-d_4).

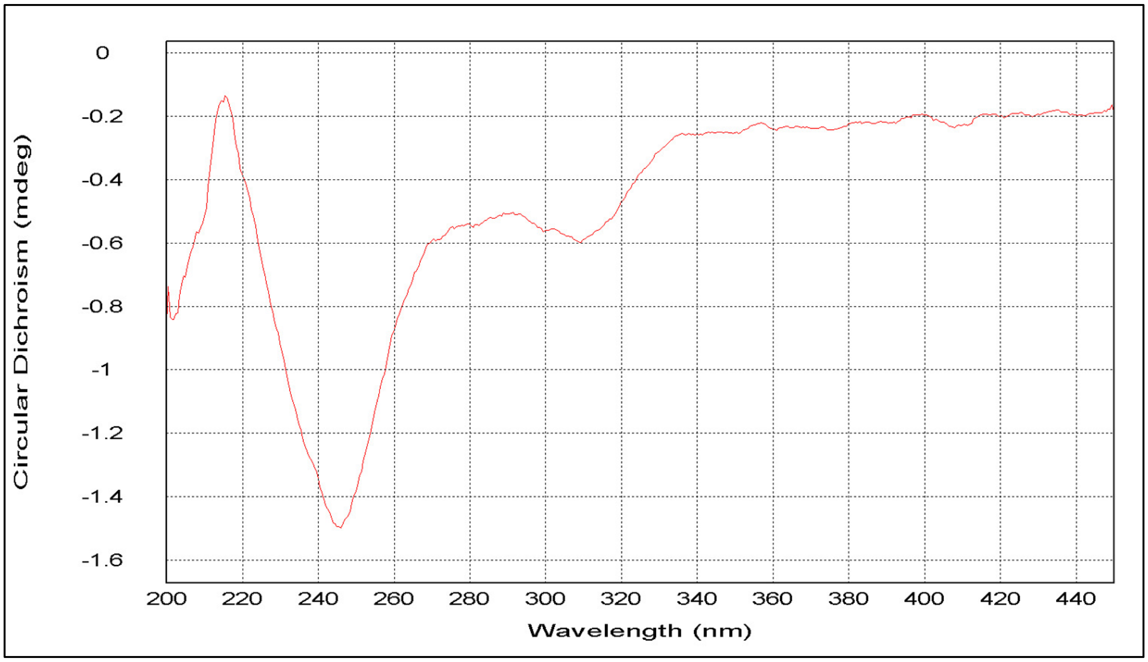

S33. ECD spectrum of compound **5** in CH_3CN at 0.2 mg/mL

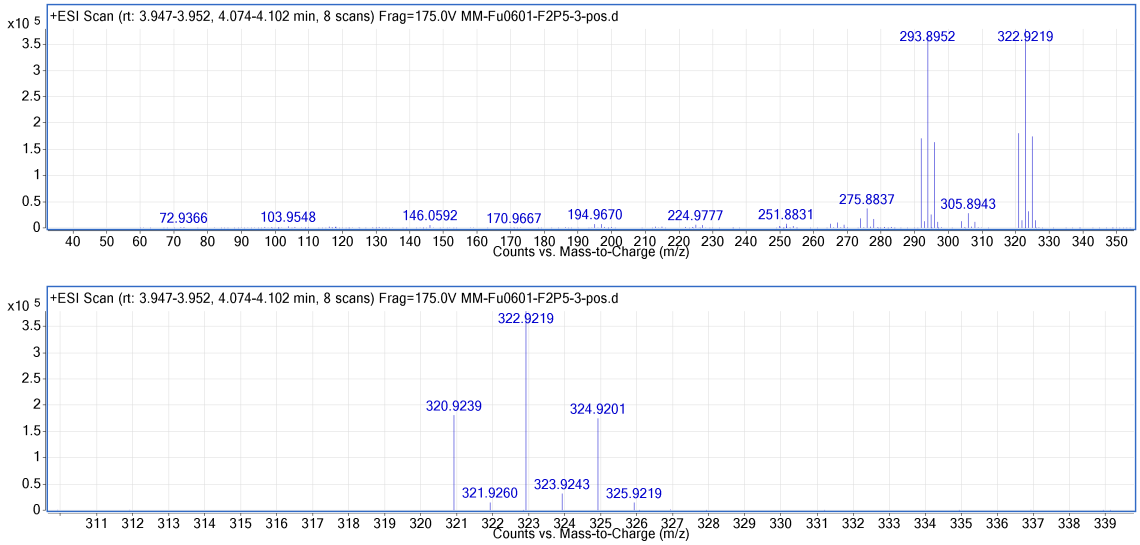

S34. ESI(+)-HRMS analysis of **6** and crop of the molecular ion.

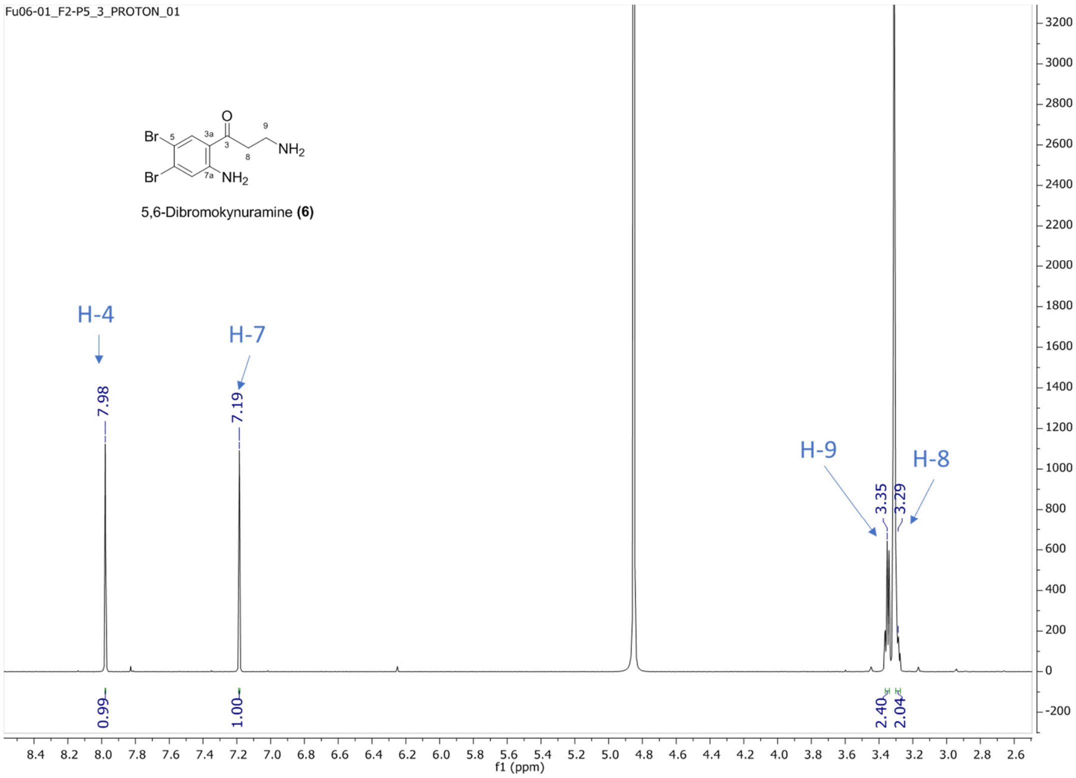

S35. ^{1}H NMR spectrum of **6** (500 MHz, MeOH-d_4).

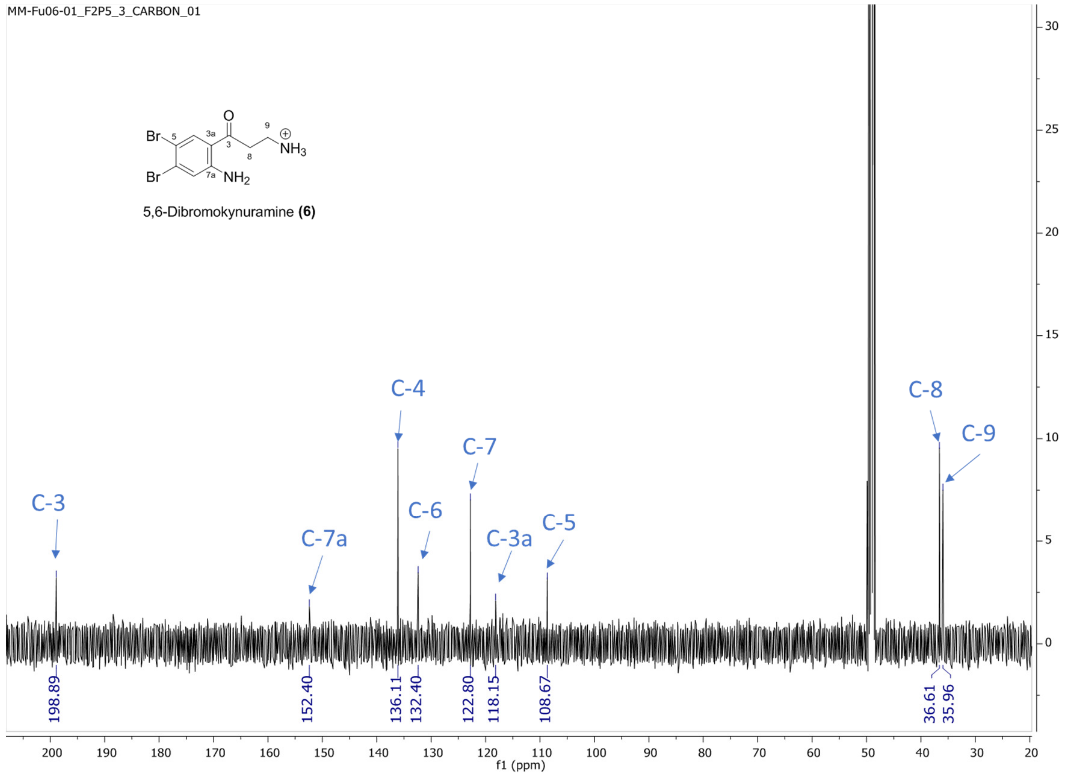

S36. ^{13}C NMR spectrum of **6** (125 MHz, MeOH-d_4).

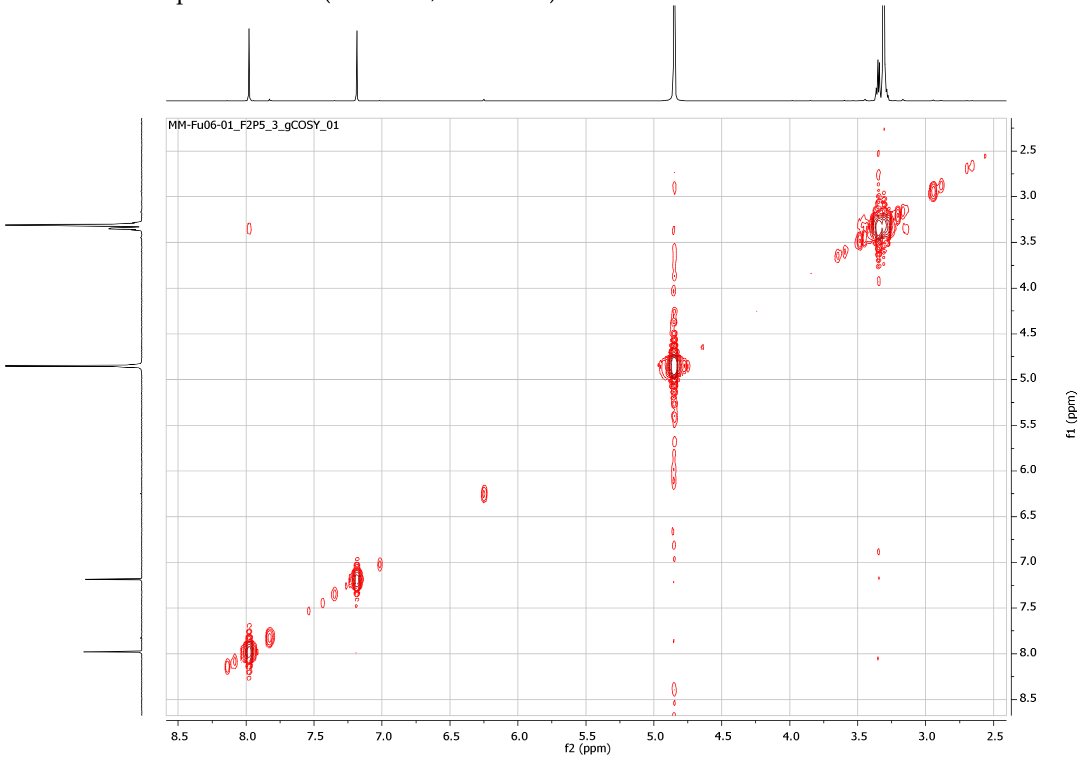

S37. COSY NMR spectrum of **6** (500 MHz, MeOH-d_4).

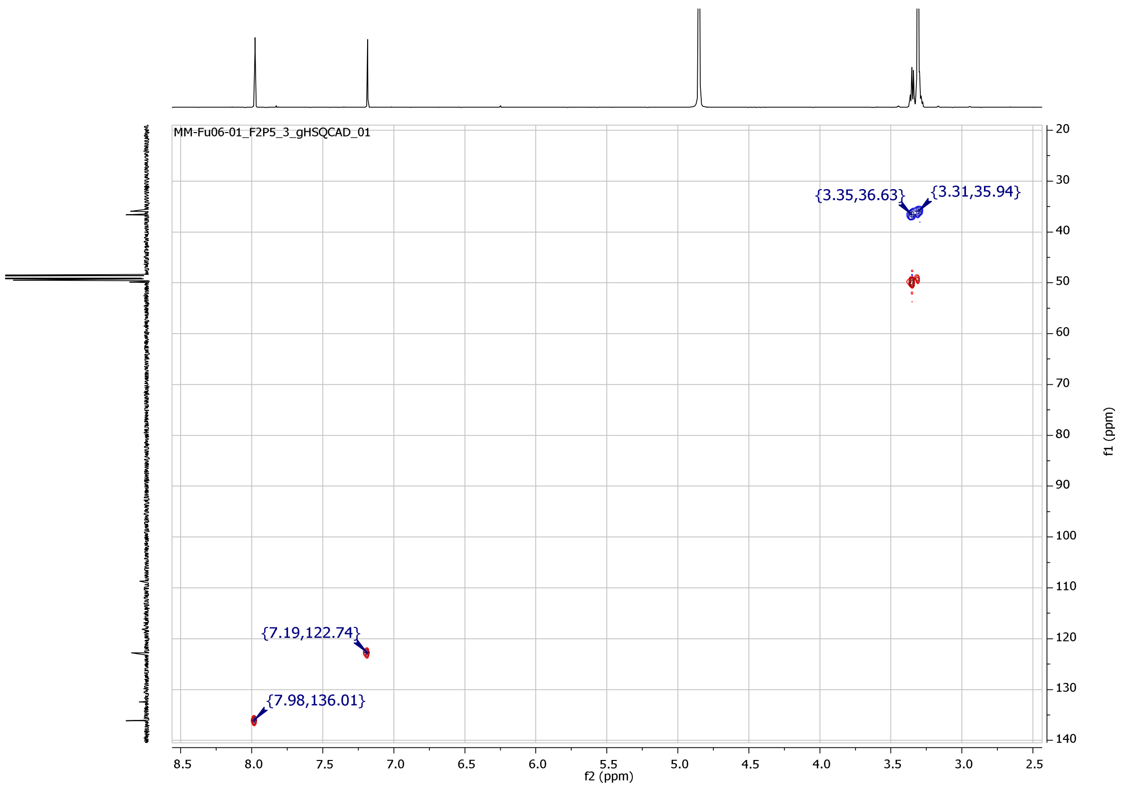

S38. HSQC NMR spectrum of **6** (500 MHz, MeOH-d_4).

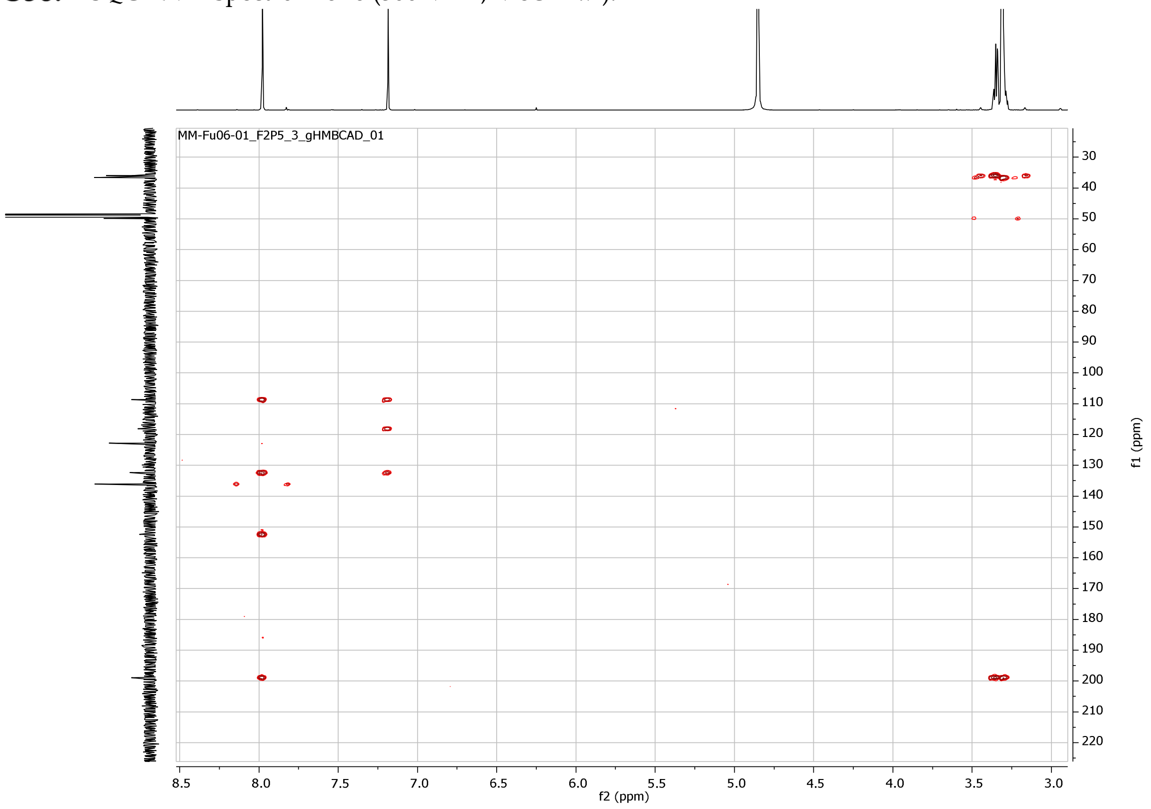

S39. HMBC NMR spectrum of **6** (500 MHz, MeOH-d_4).

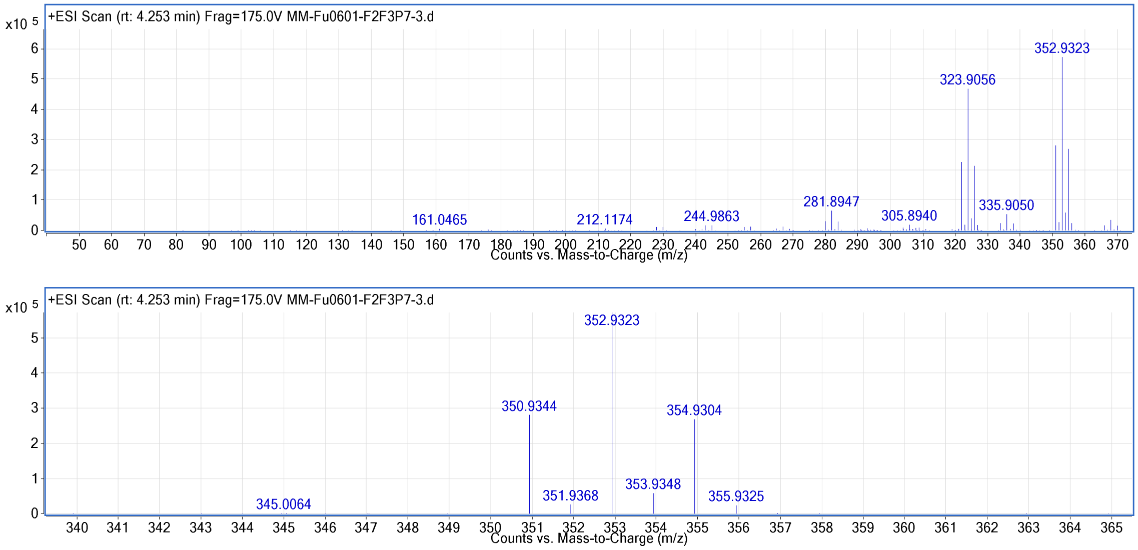

S40. ESI(+)-HRMS analysis of **7** and crop of the molecular ion.

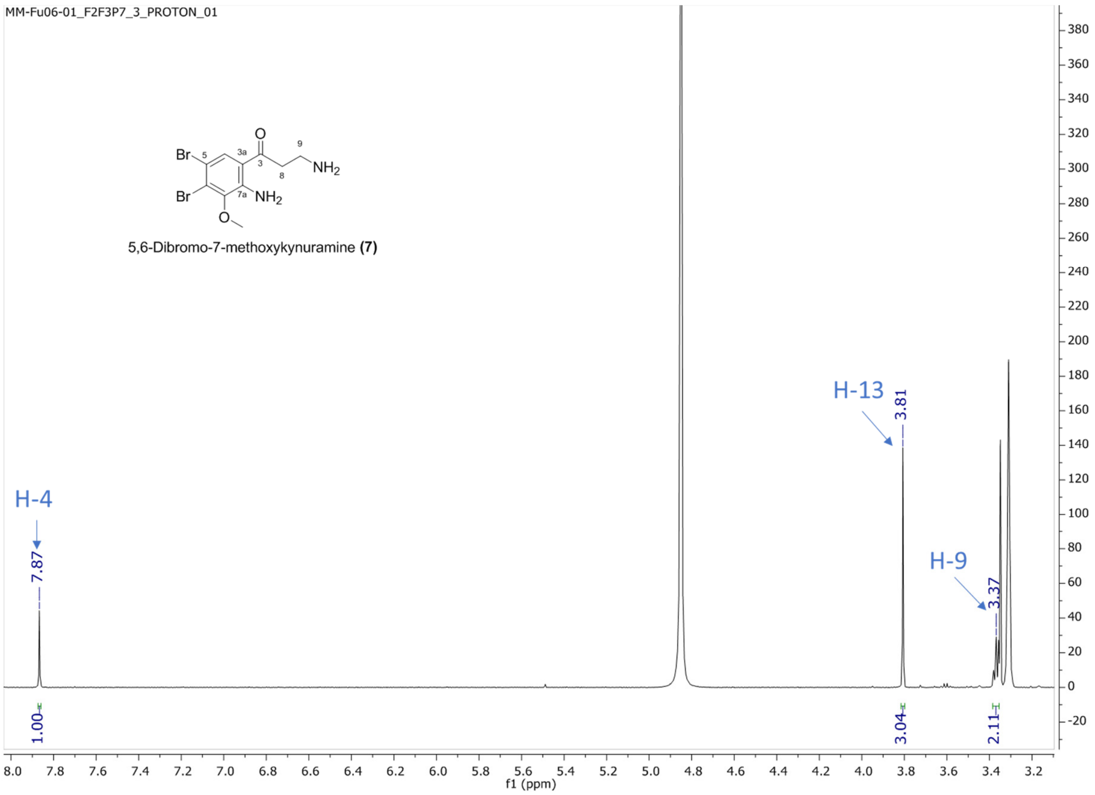

S41. ^{1}H NMR spectrum of **7** (500 MHz, MeOH-d_4).

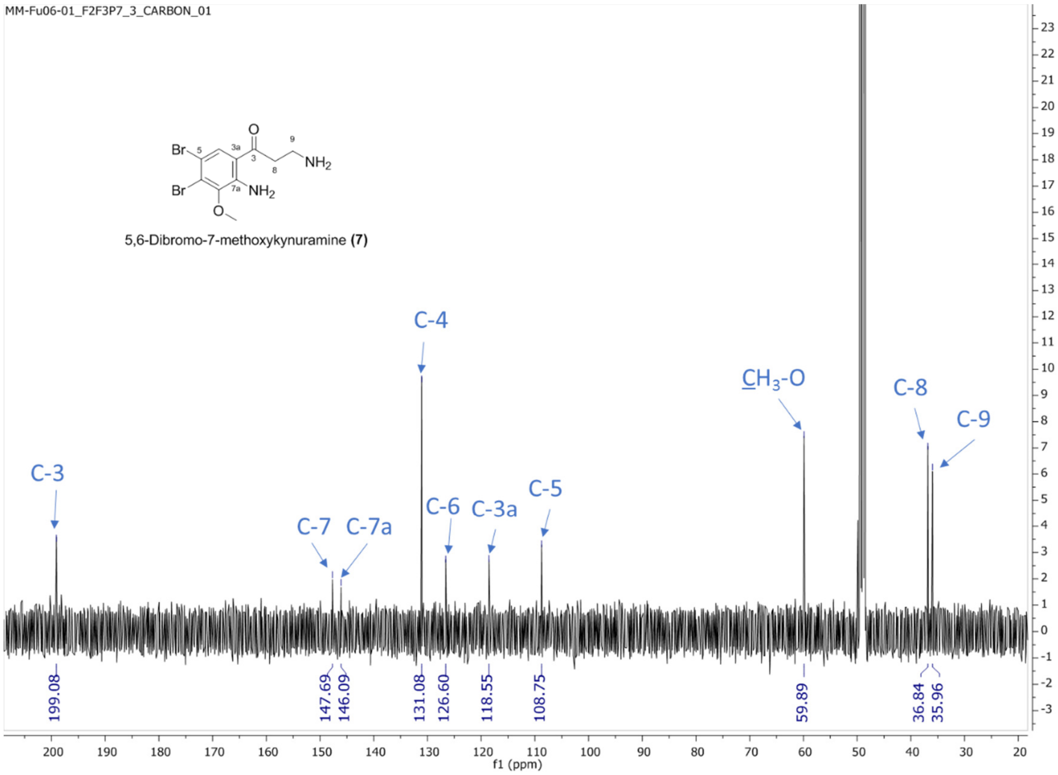

S42. ^{13}C NMR spectrum of **7** (125 MHz, MeOH-d_4).

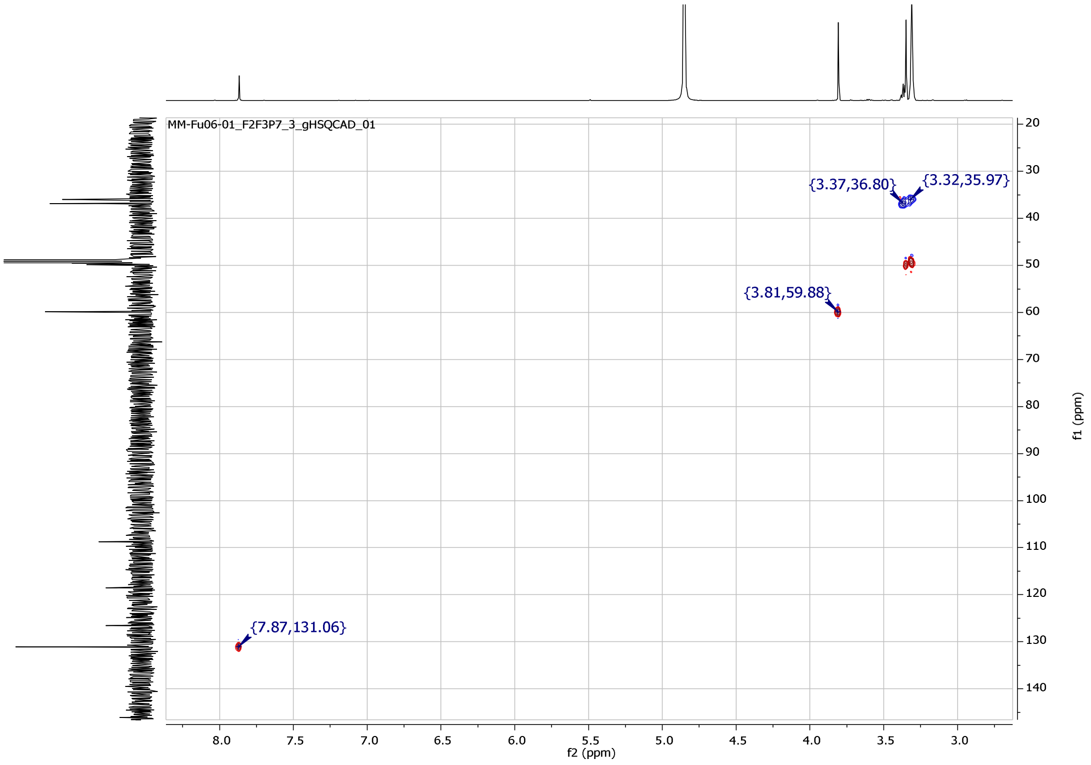

S43. HSQC NMR spectrum of **7** (500 MHz, MeOH-d_4).

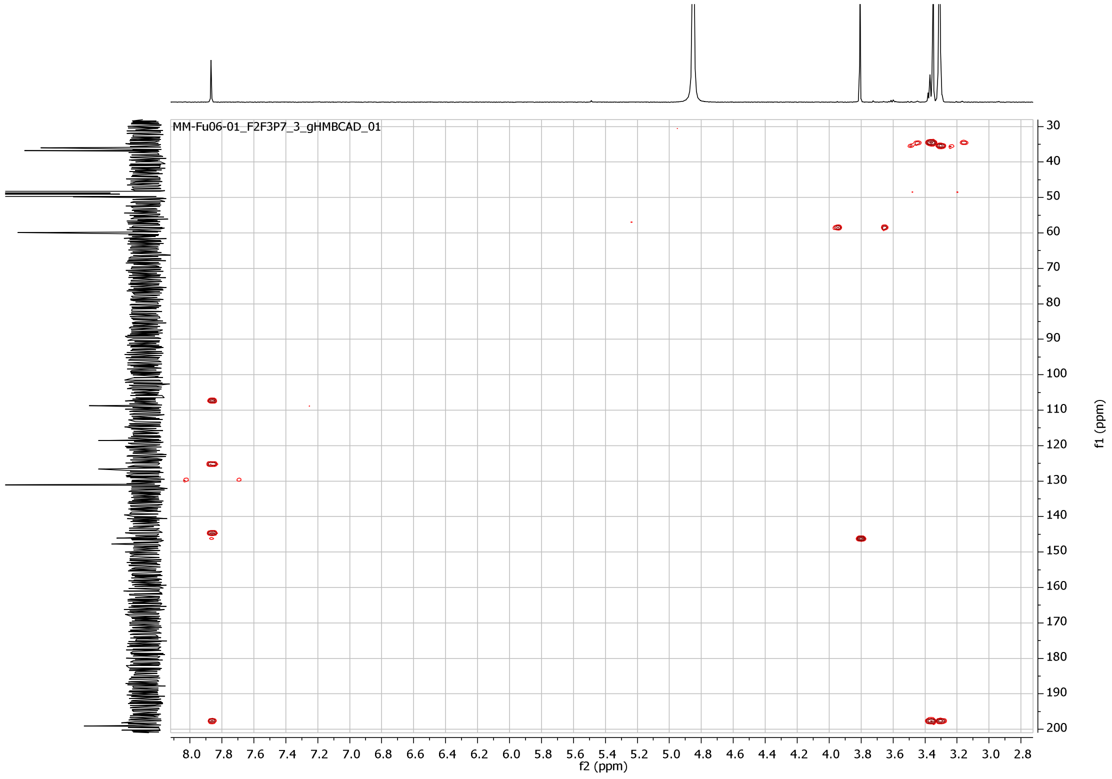

S44. HMBC NMR spectrum of **7** (500 MHz, MeOH-d_4).

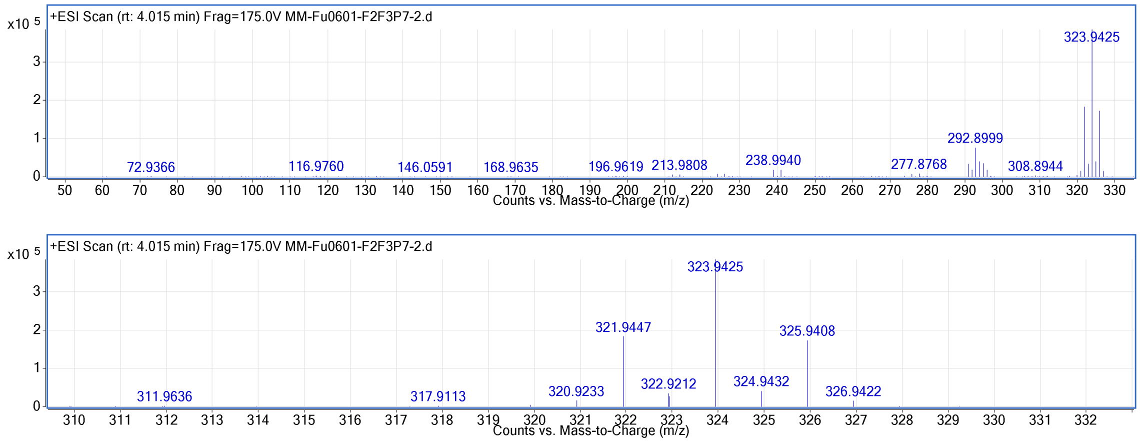

S45. ESI(+)-HRMS analysis of **8** and crop of the molecular ion.

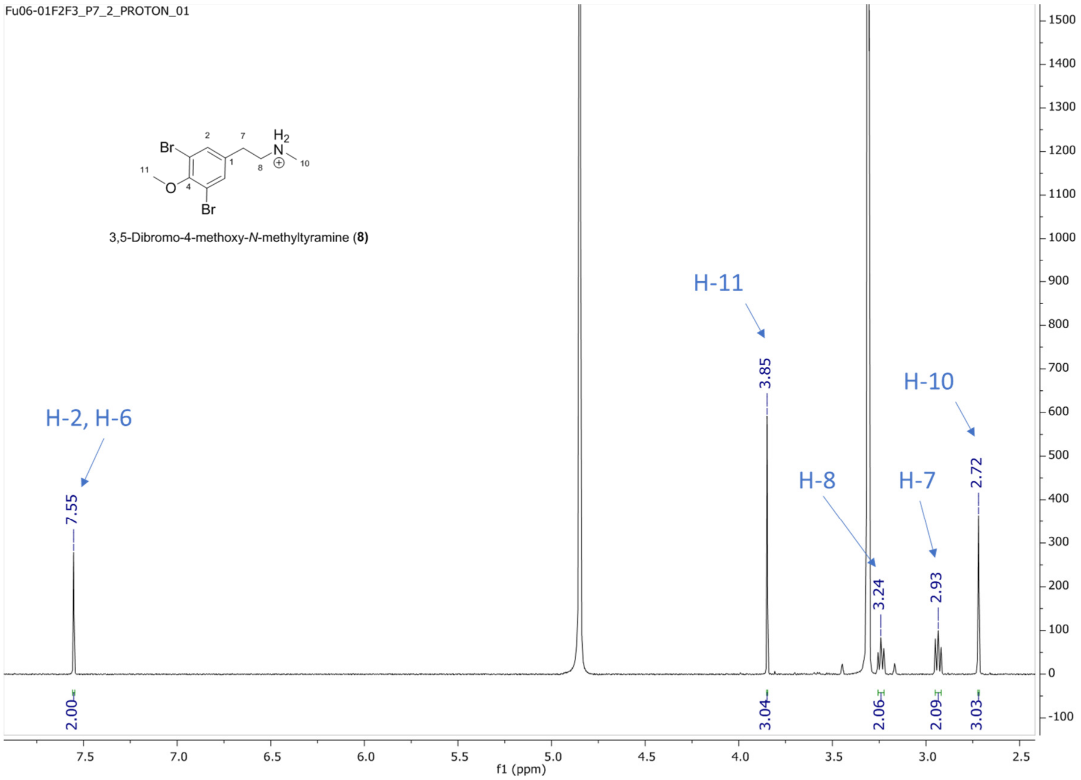

S46. ^{1}H NMR spectrum of **8** (500 MHz, MeOH-d_4).

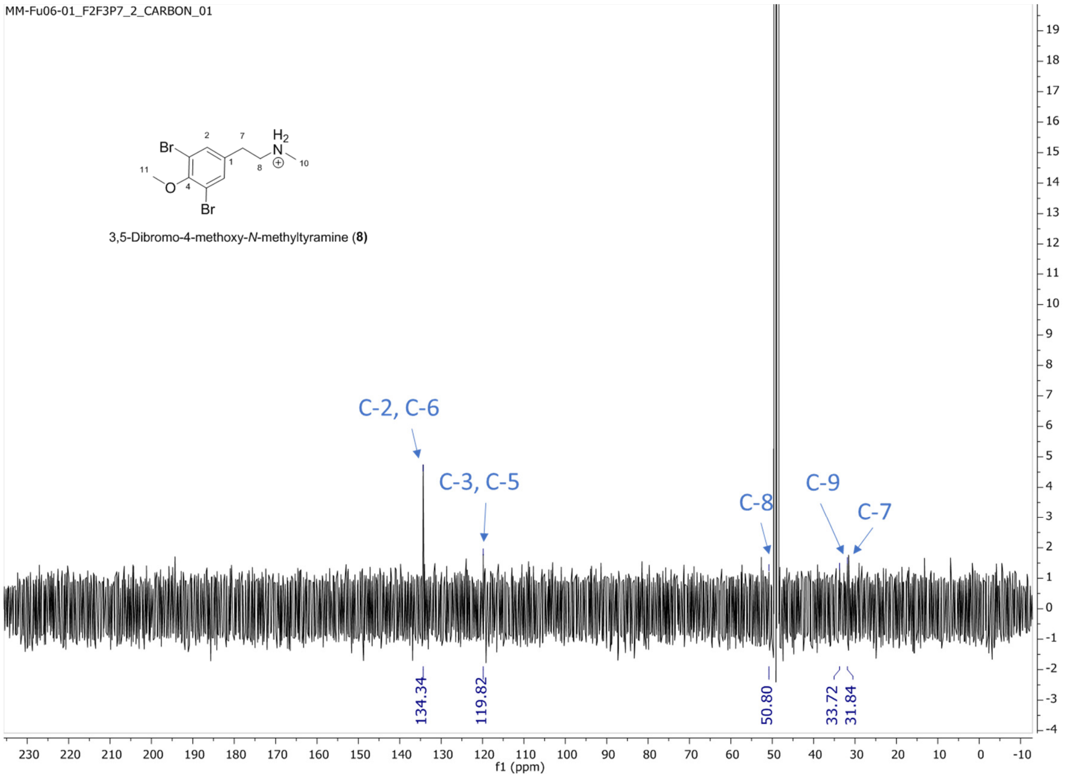

S47. ^{13}C NMR spectrum of **8** (125 MHz, MeOH-d_4).

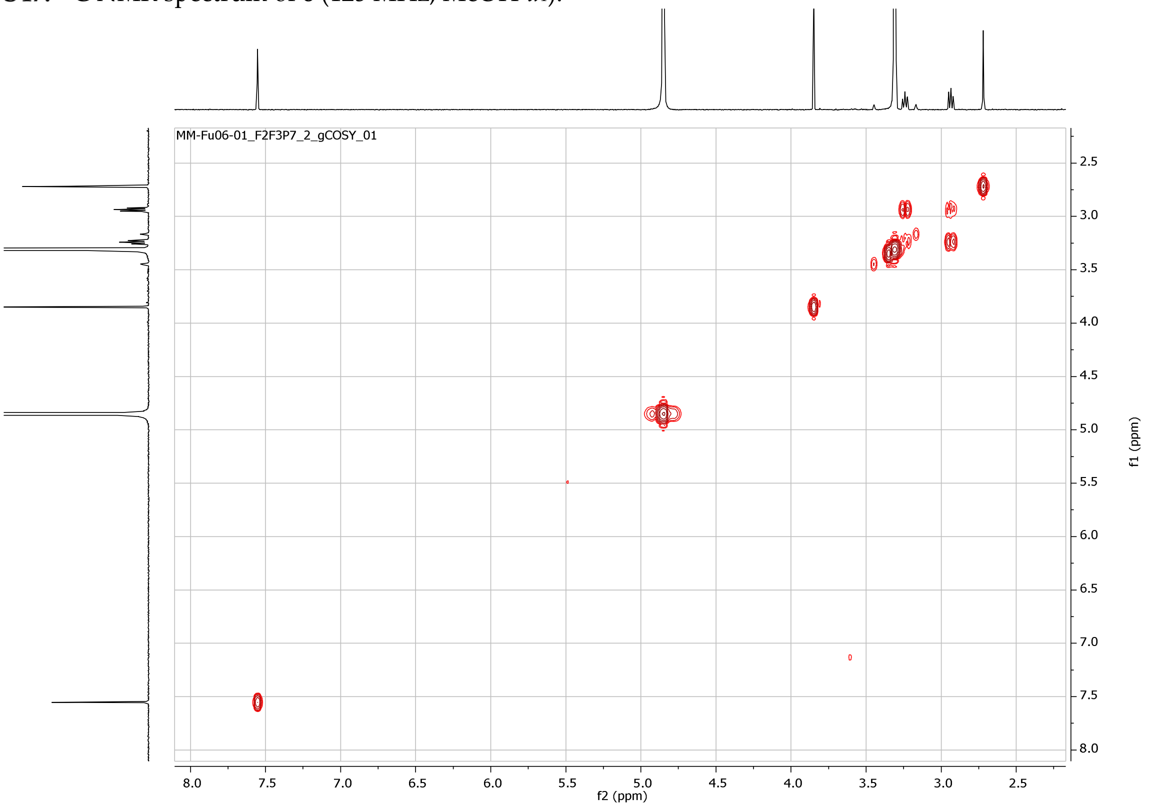

S48. COSY NMR spectrum of **8** (500 MHz, MeOH-d_4).

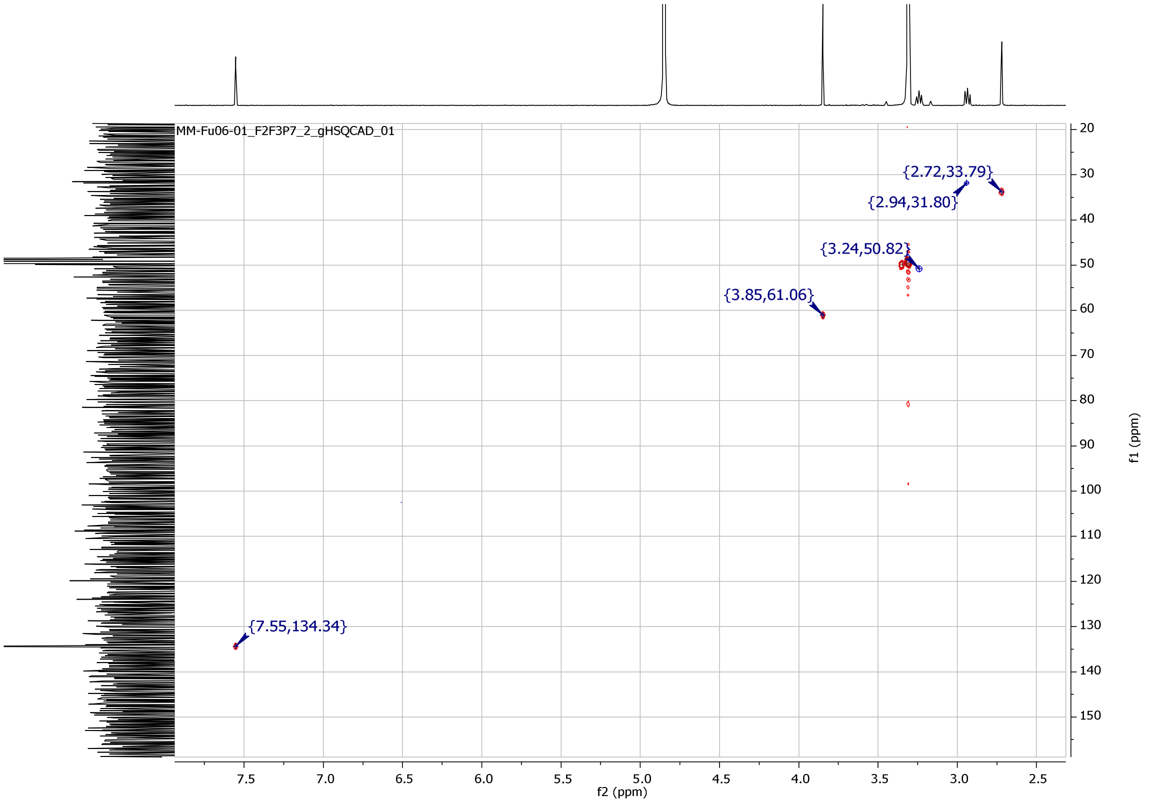

S49. HSQC NMR spectrum of **8** (500 MHz, MeOH-d_4).

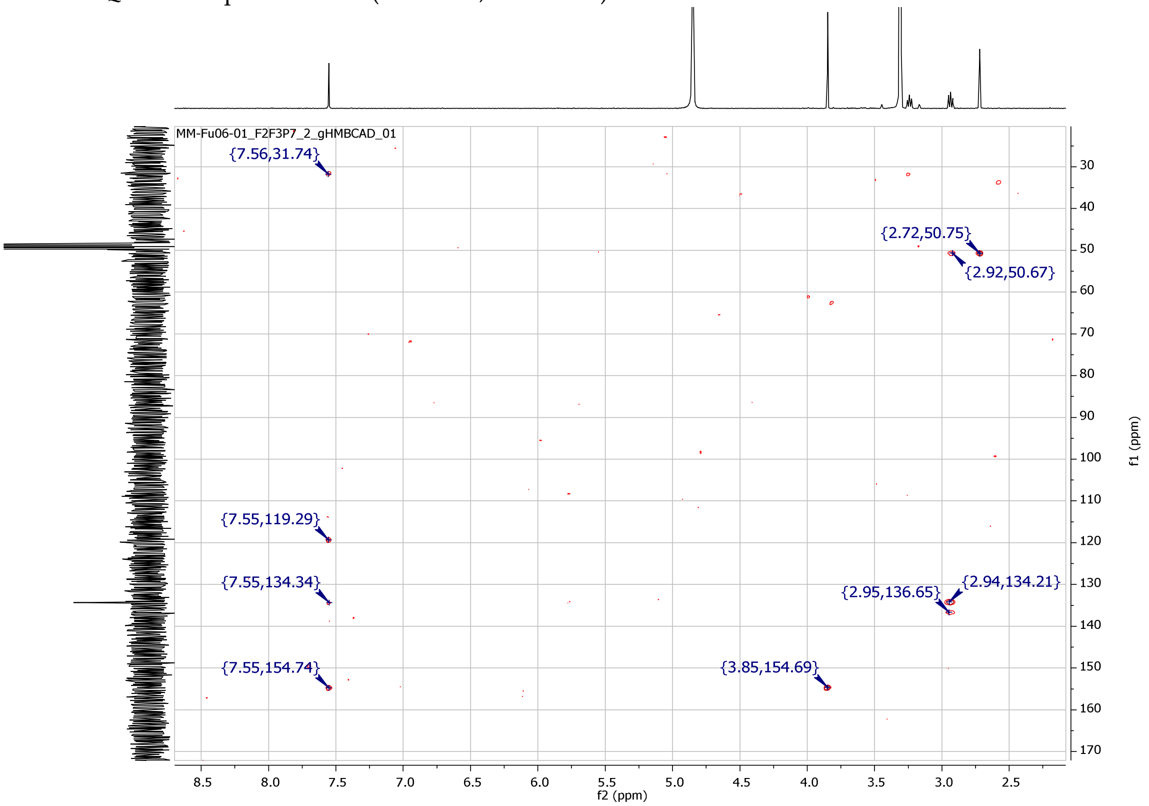

S50. HMBC NMR spectrum of **8** (500 MHz, MeOH-d_4).

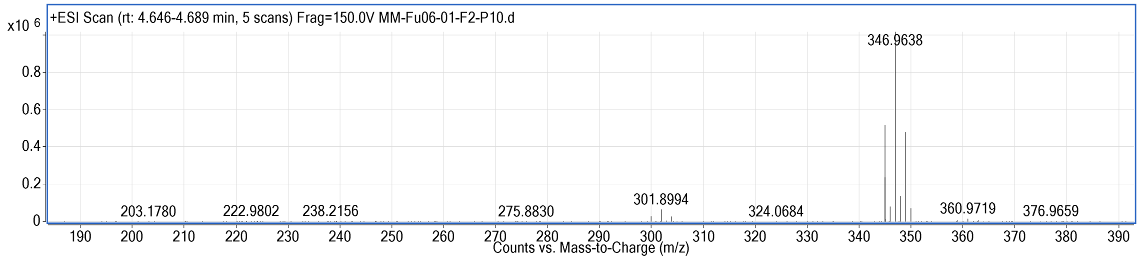

S51. ESI(+)-HRMS analysis of **9**

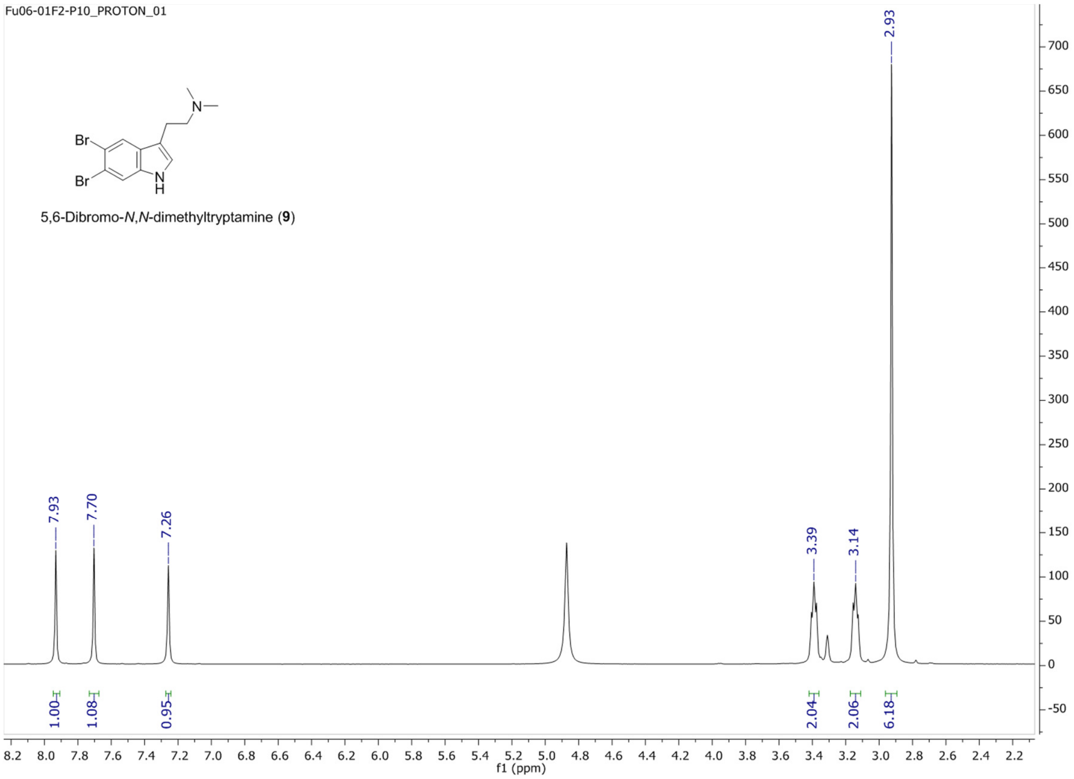

S52. ^{1}H NMR spectrum of **9** (500 MHz, MeOH-d_4).

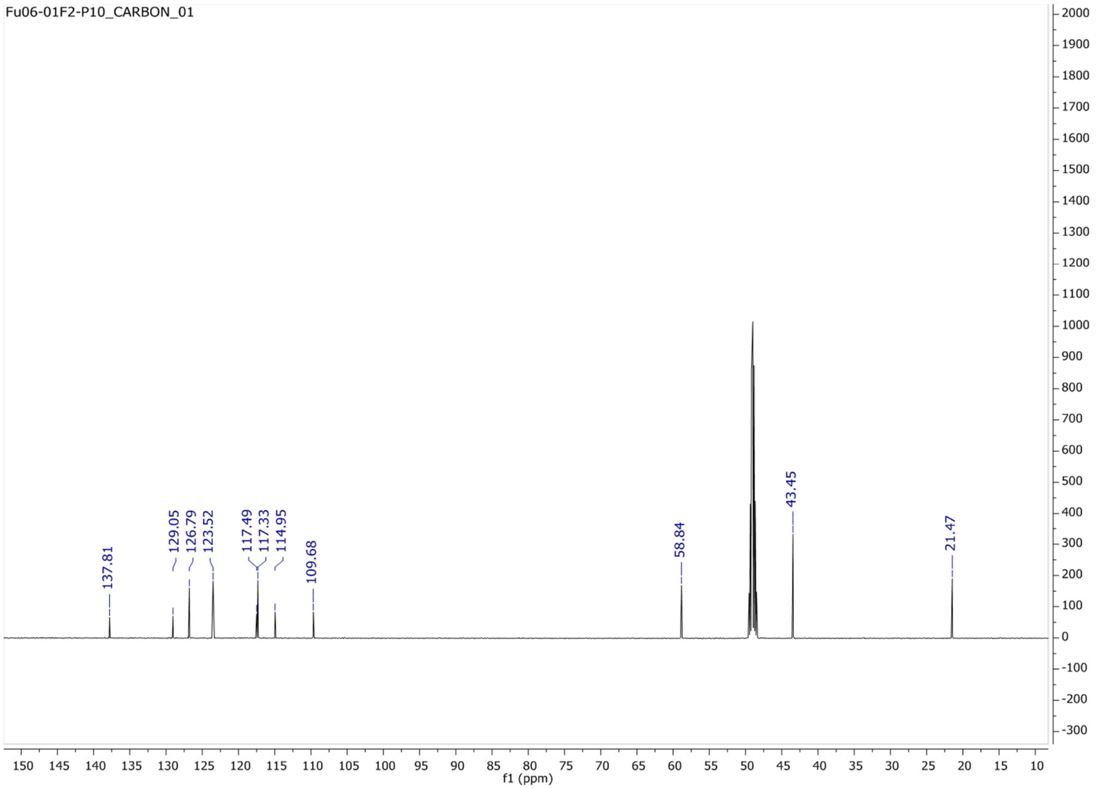

S53. ^{13}C NMR spectrum of **9** (125 MHz, MeOH-*d*$_4$).

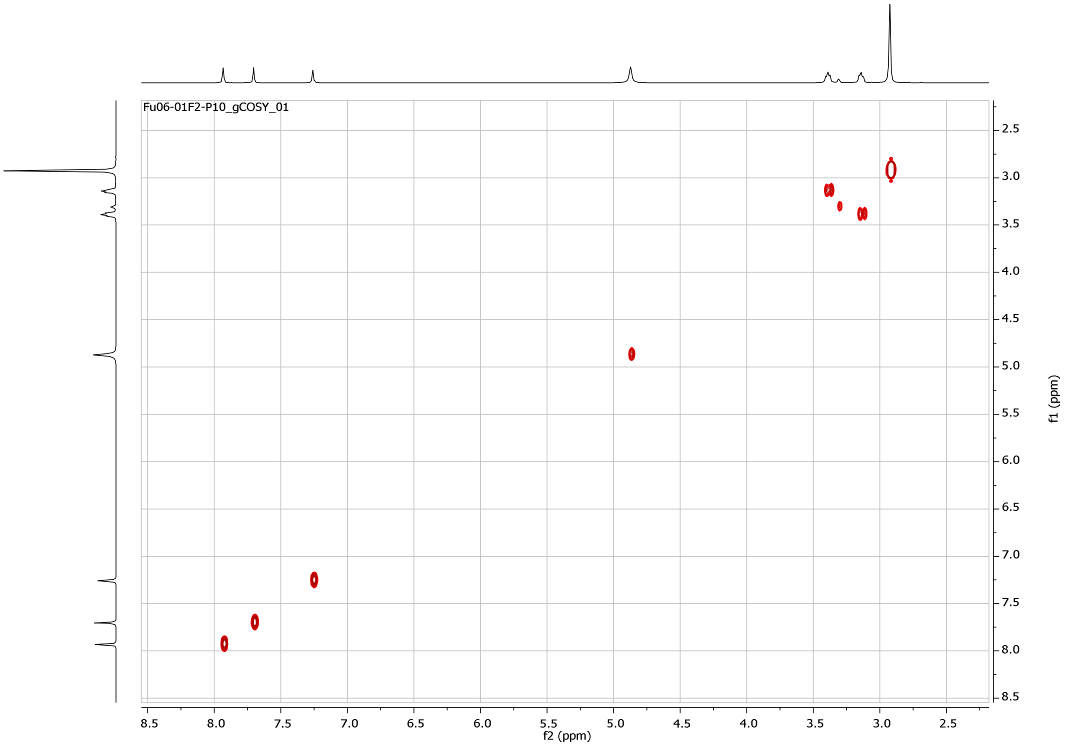

S54. COSY NMR spectrum of **9** (500 MHz, MeOH-*d*$_4$).

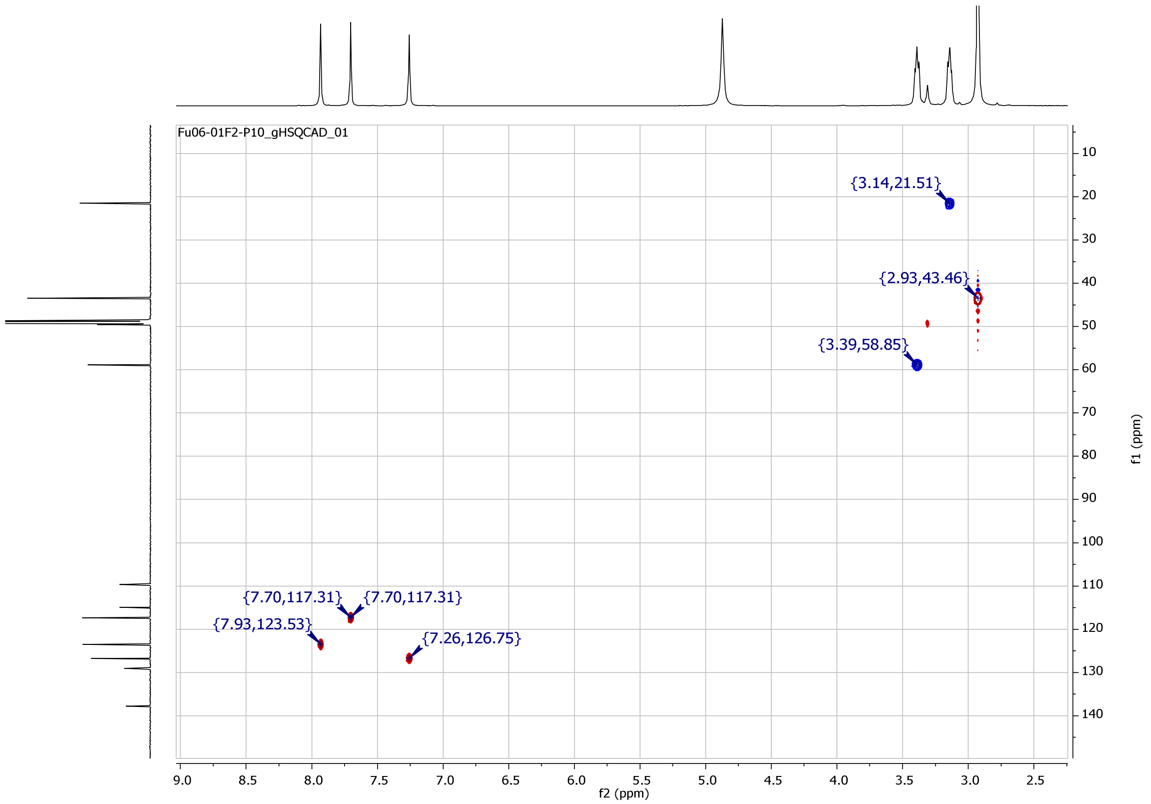

S55. HSQC NMR spectrum of **9** (500 MHz, MeOH-d_4).

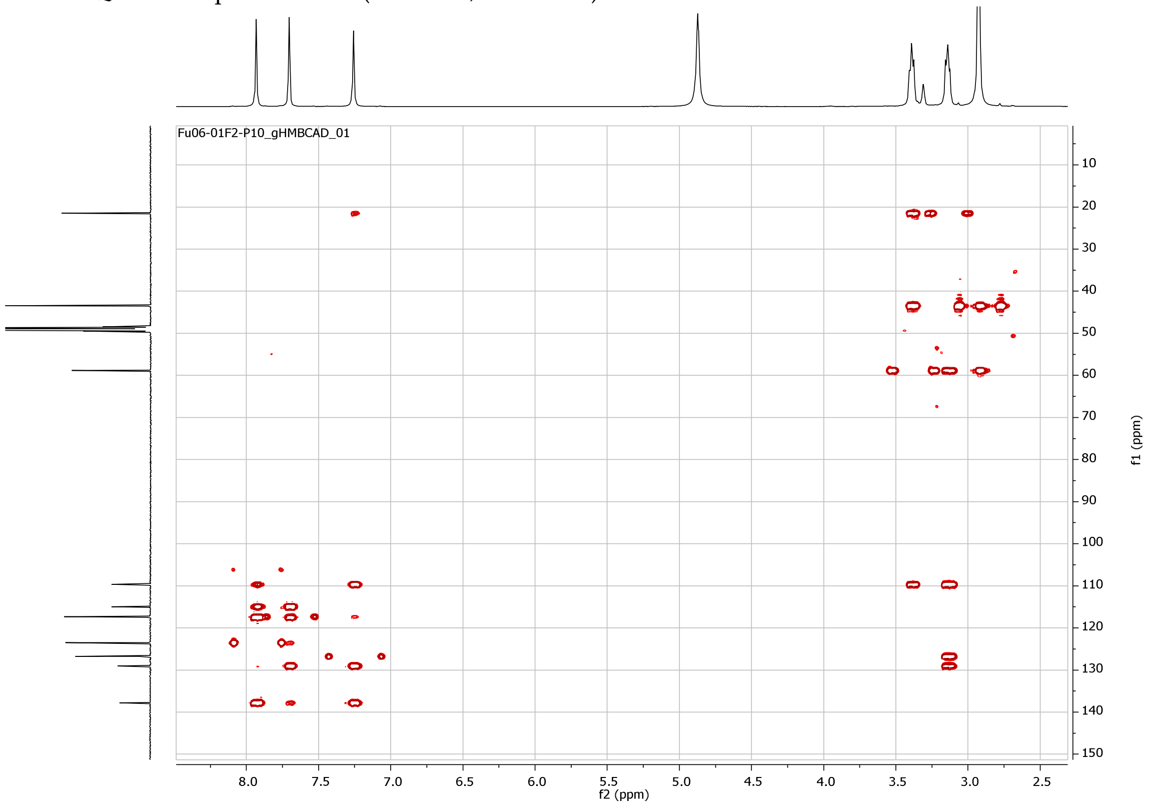

S56. HSQC NMR spectrum of **9** (500 MHz, MeOH-d_4).

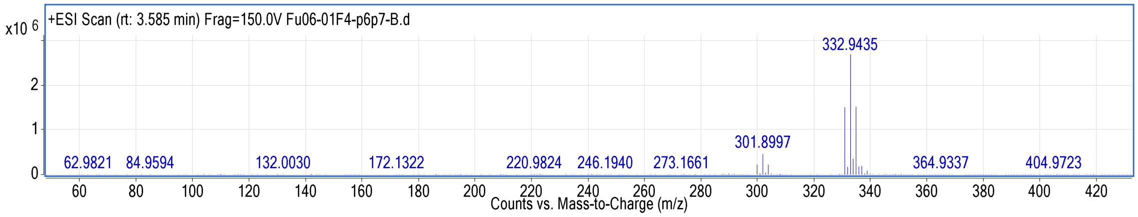

S57. ESI(+)-HRMS analysis of **10**

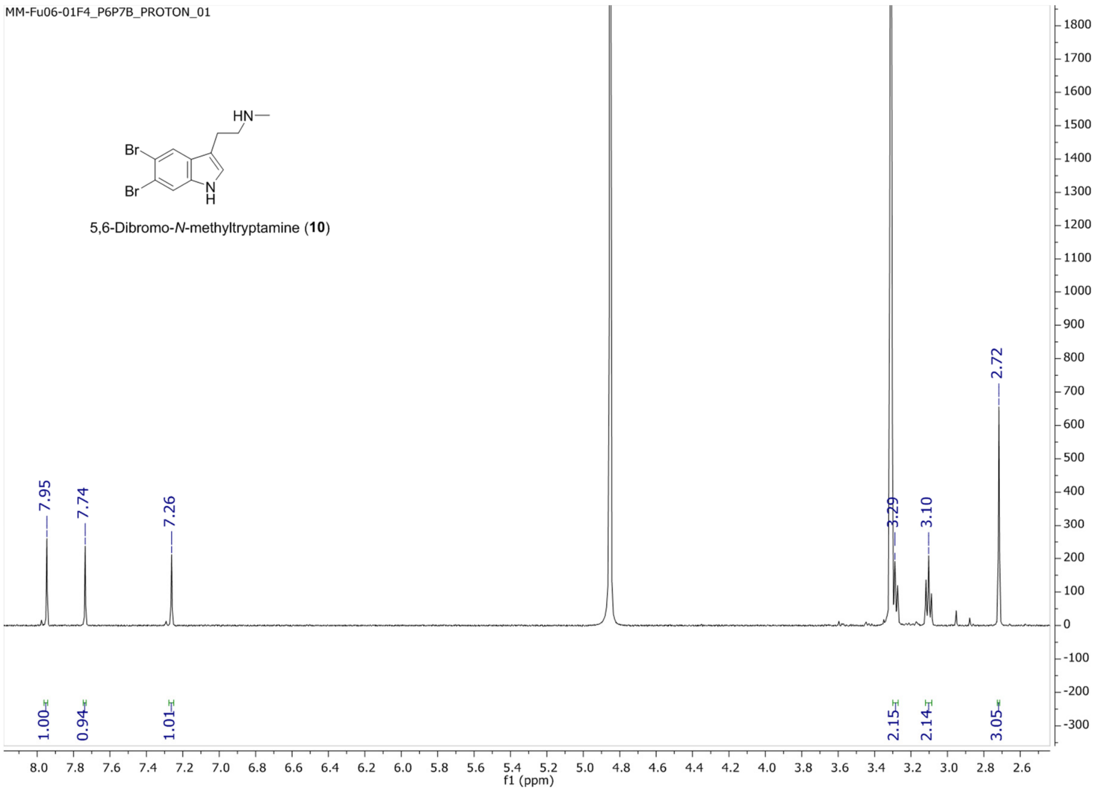

S58. ^{1}H NMR spectrum of **10** (500 MHz, MeOH-d_4).

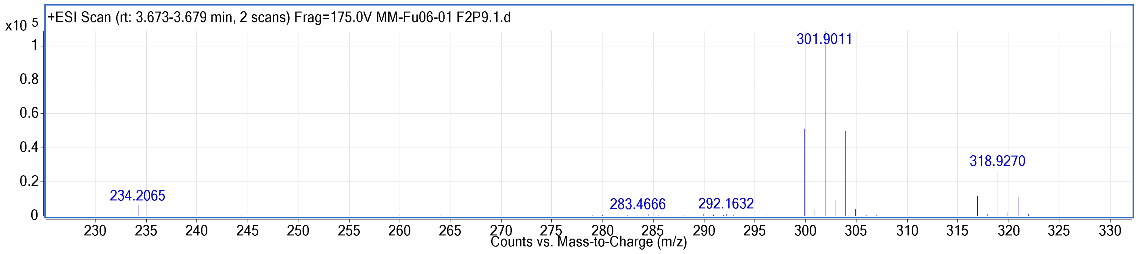

S59. ESI(+)-HRMS analysis of **11**

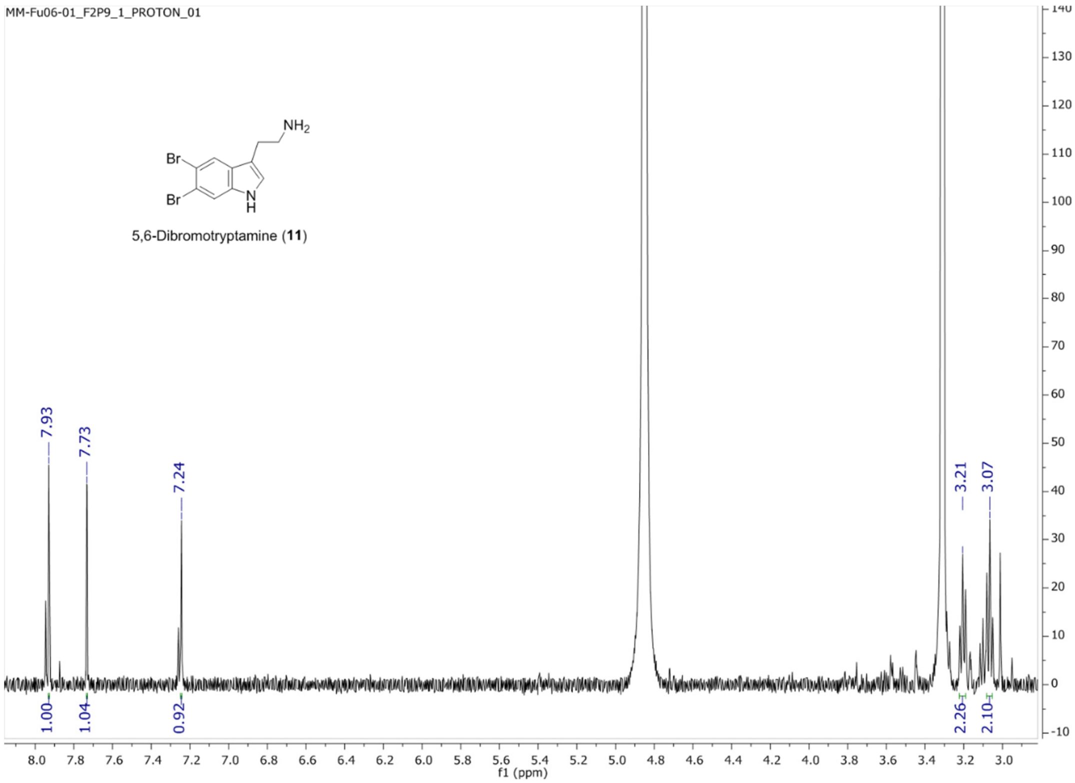

S60. ^{1}H NMR spectrum of **11** (500 MHz, MeOH-d_4).

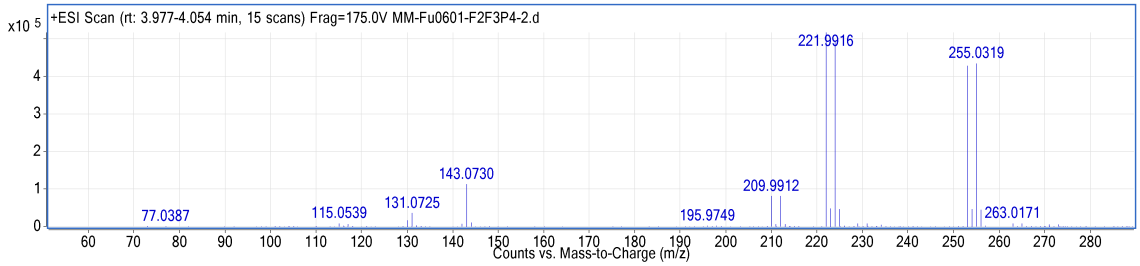

S61. ESI(+)-HRMS analysis of **12**

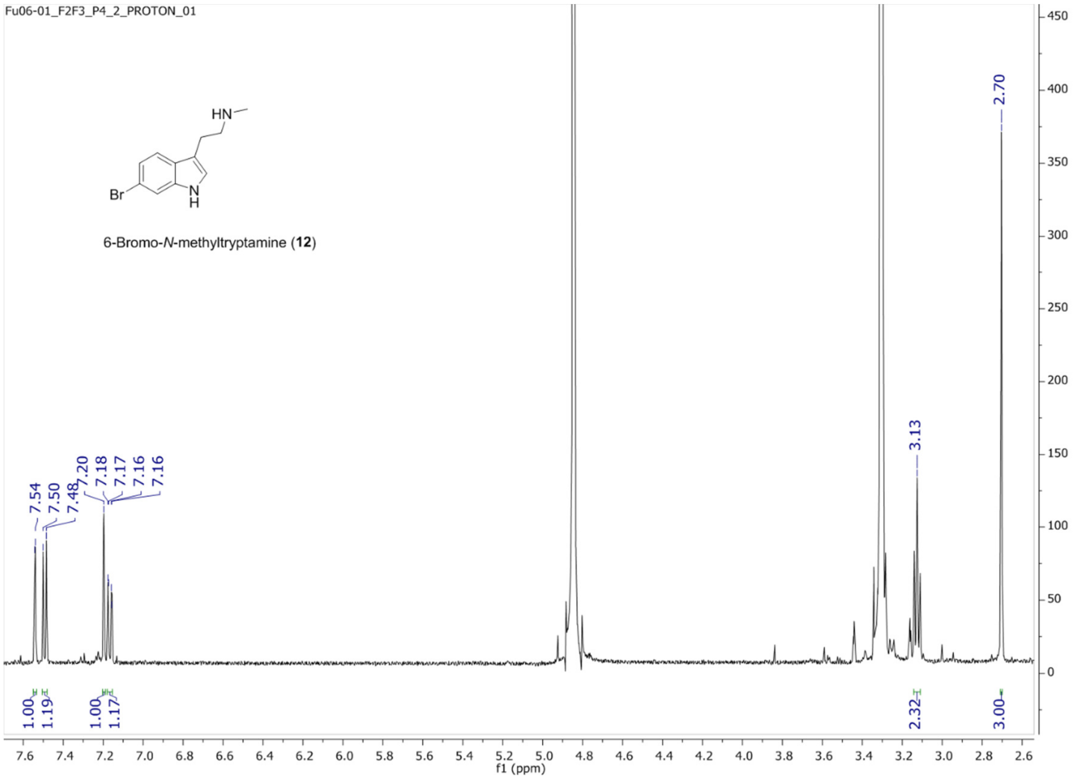

S62. ^{1}H NMR spectrum of **12** (500 MHz, MeOH-*d₄*).

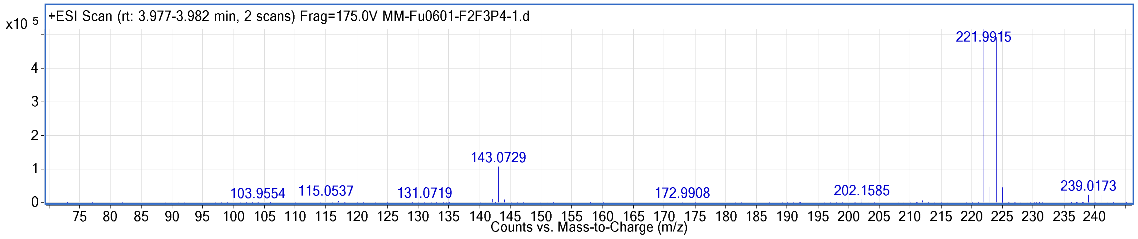

S63. ESI(+)-HRMS analysis of **13**

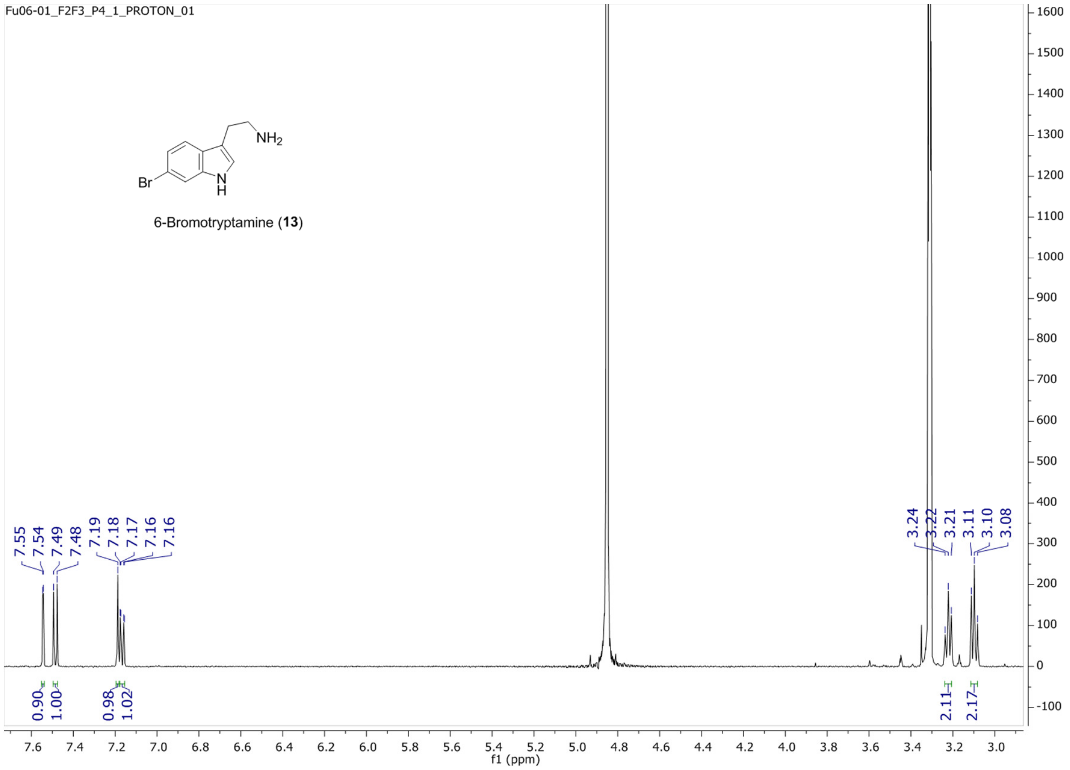

S64. ^{1}H NMR spectrum of **13** (500 MHz, MeOH-d_4).

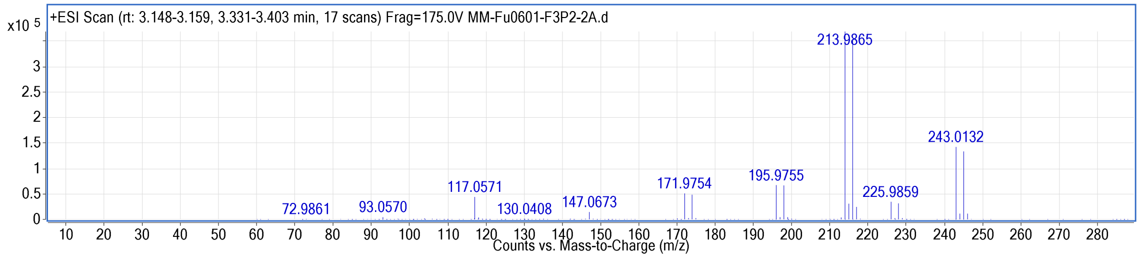

S65. ESI(+)-HRMS analysis of **14**

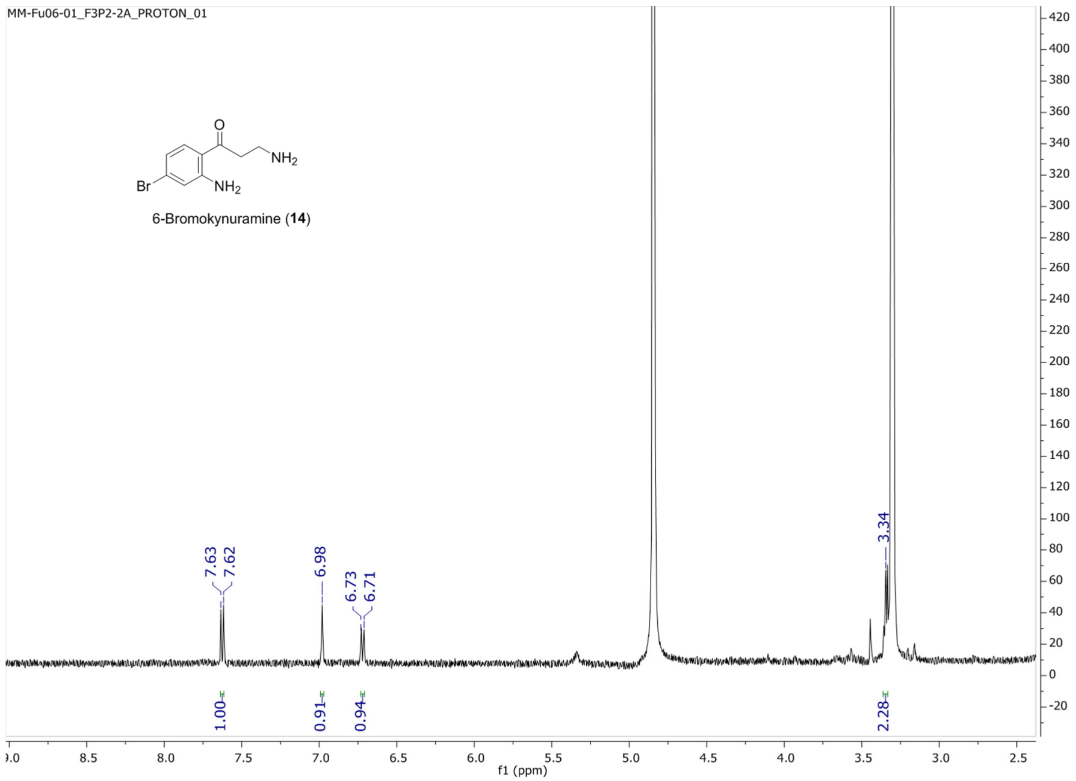

S66. ^{1}H NMR spectrum of **14** (500 MHz, MeOH-d_4).

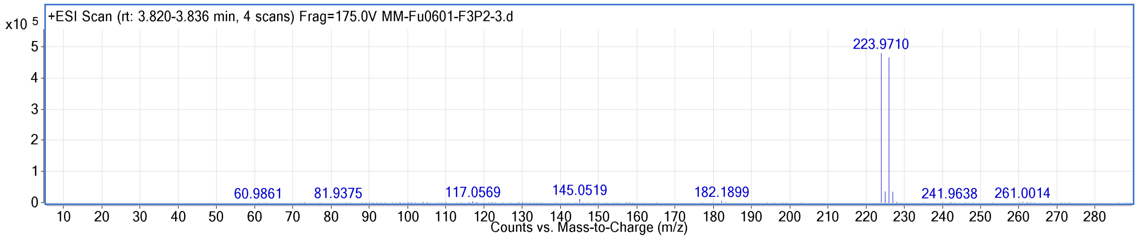

S67. ESI(+)-HRMS analysis of **15**

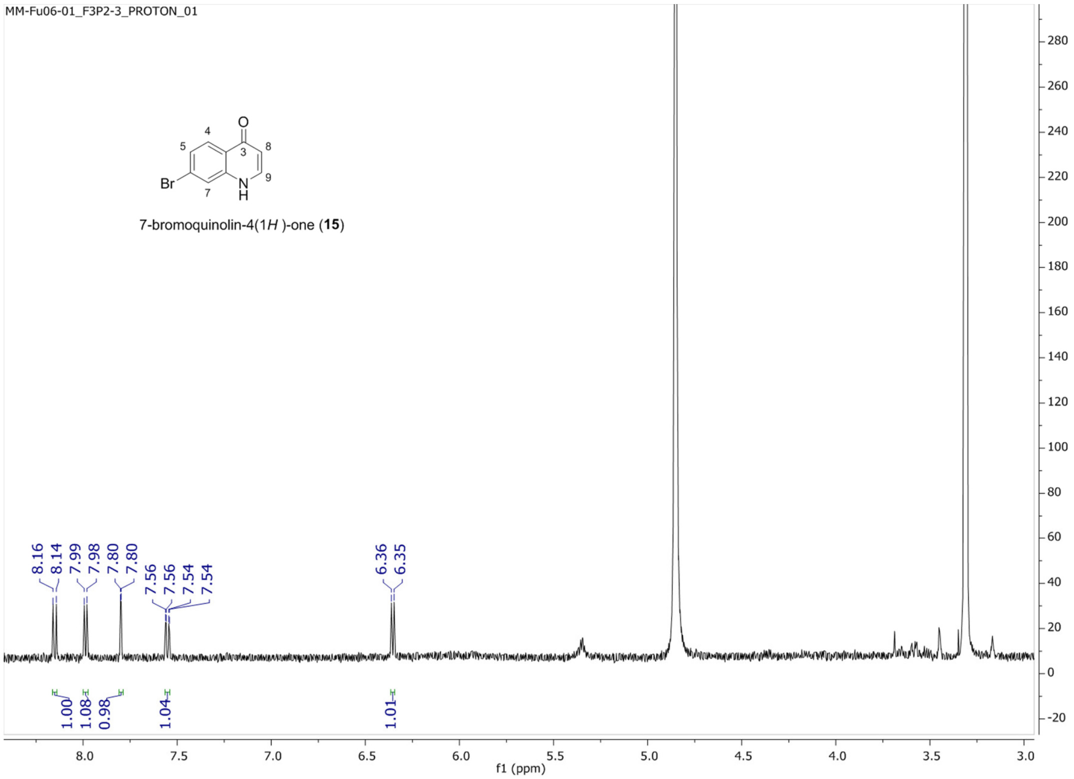

S68. ^{1}H NMR spectrum of **15** (500 MHz, MeOH-d_4).

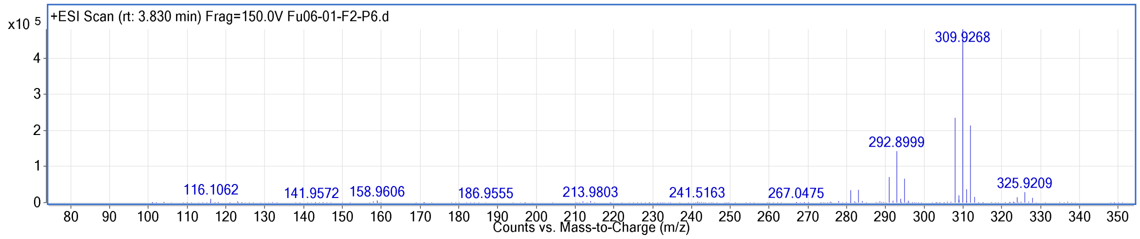

S69. ESI(+)-HRMS analysis of **16**

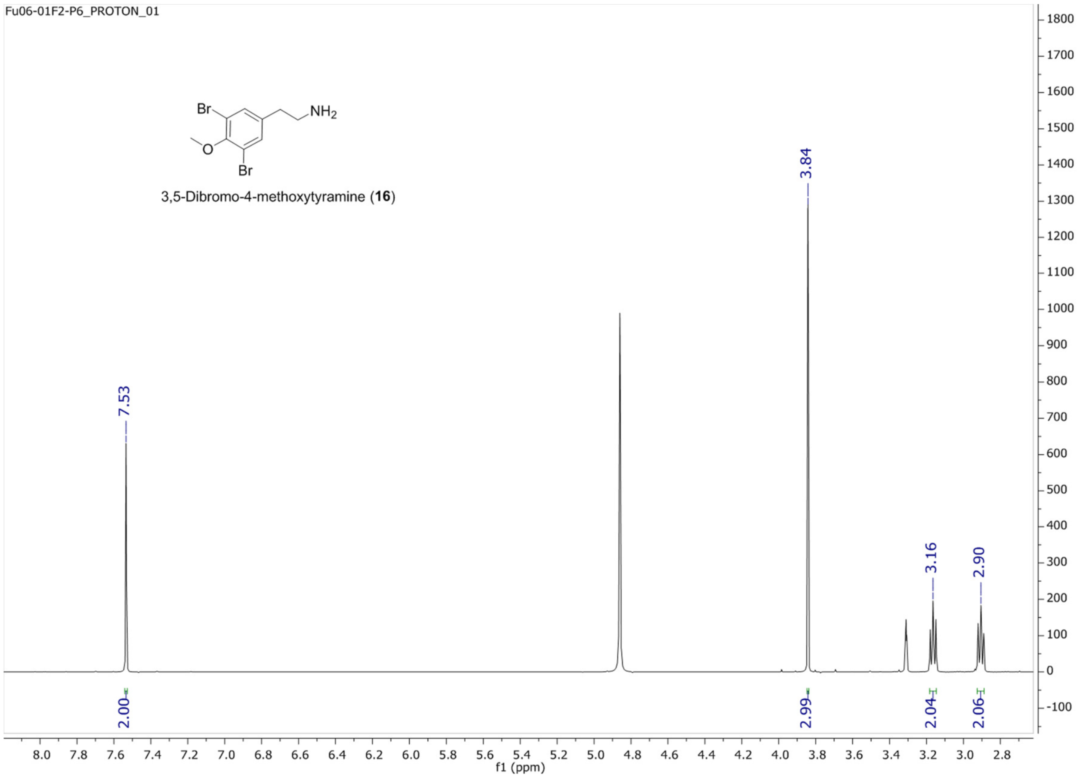

S70. ¹H NMR spectrum of **16** (500 MHz, MeOH-d_4).

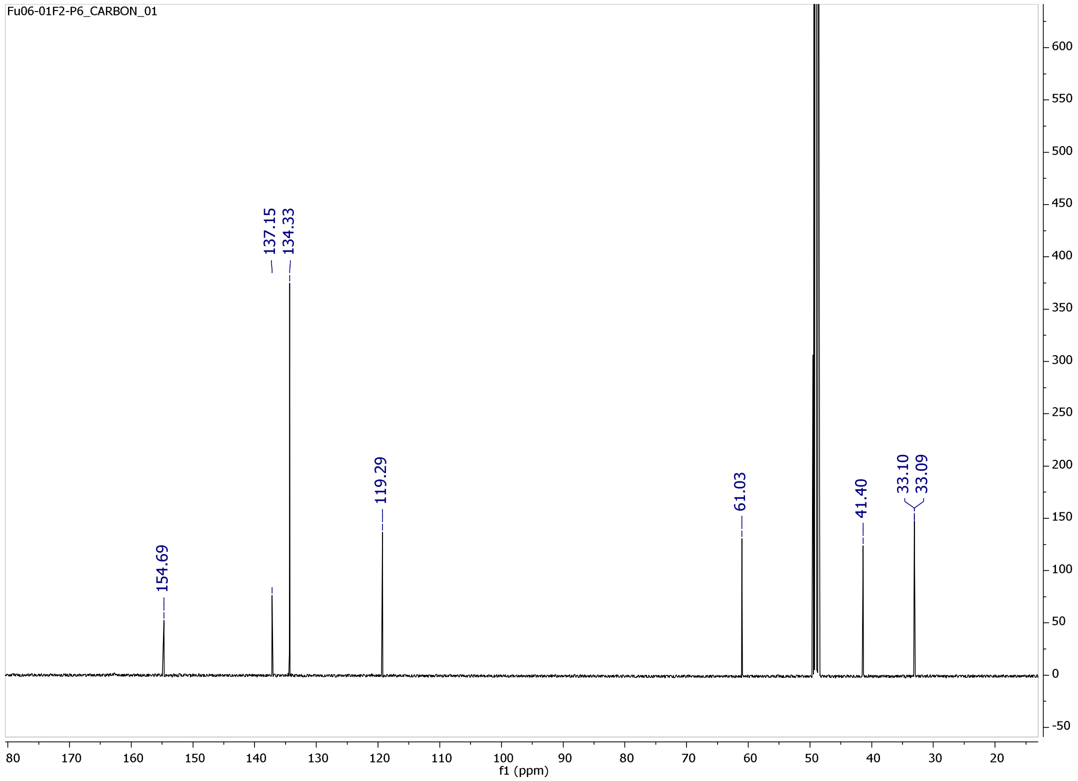

S71. ^{13}C NMR spectrum of **16** (125 MHz, MeOH-d_4).

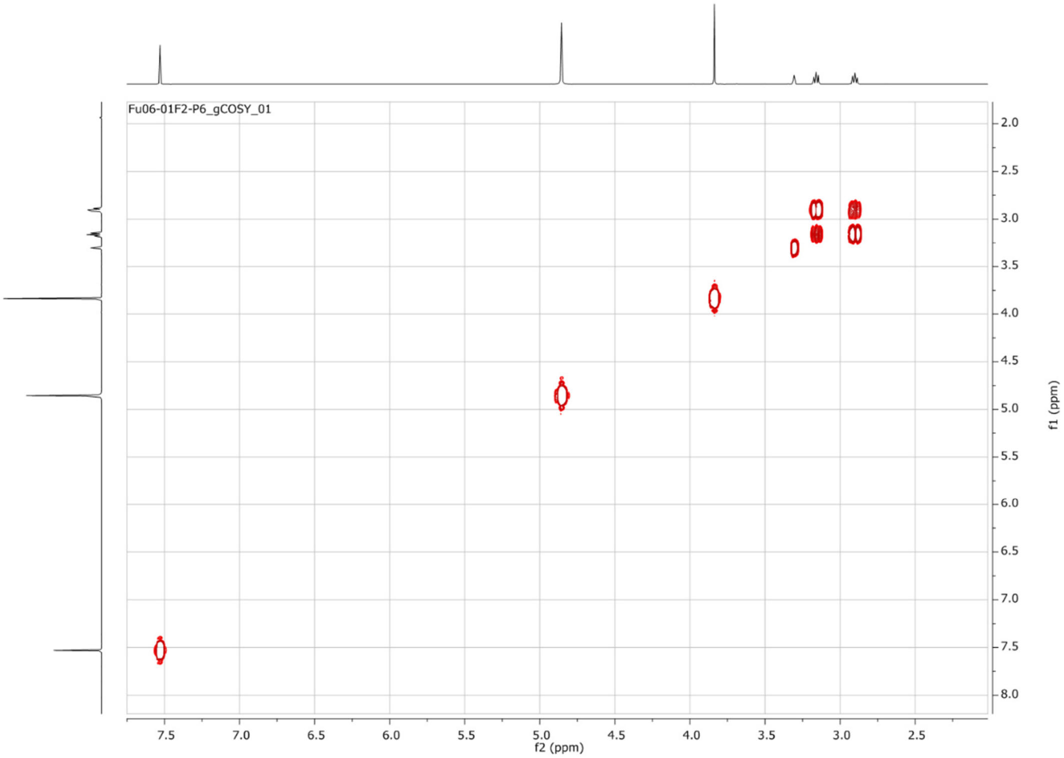

S72. COSY NMR spectrum of **16** (500 MHz, MeOH-d_4).

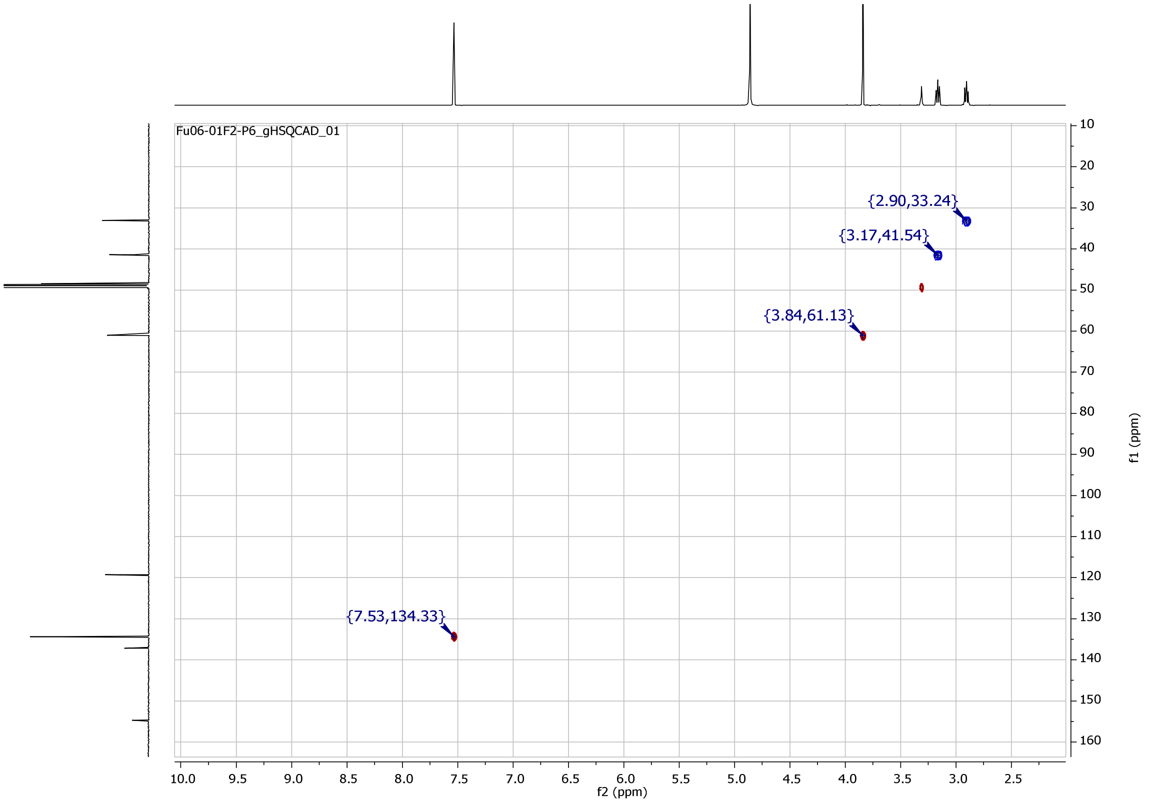

S73. HSQC NMR spectrum of **16** (500 MHz, MeOH-*d₄*).

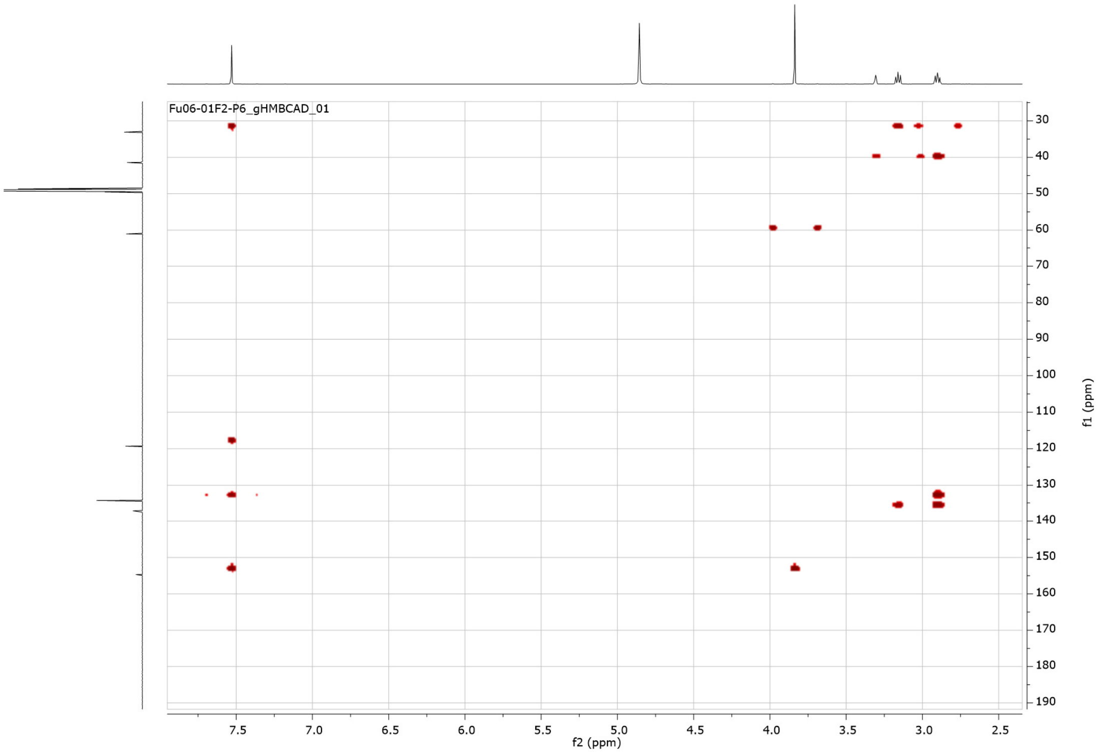

S74. HMBC NMR spectrum of **16** (500 MHz, MeOH-*d₄*).

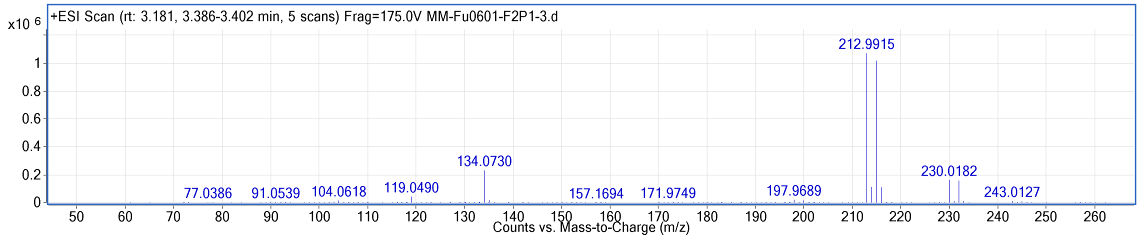

S75. ESI(+)-HRMS analysis of **17**

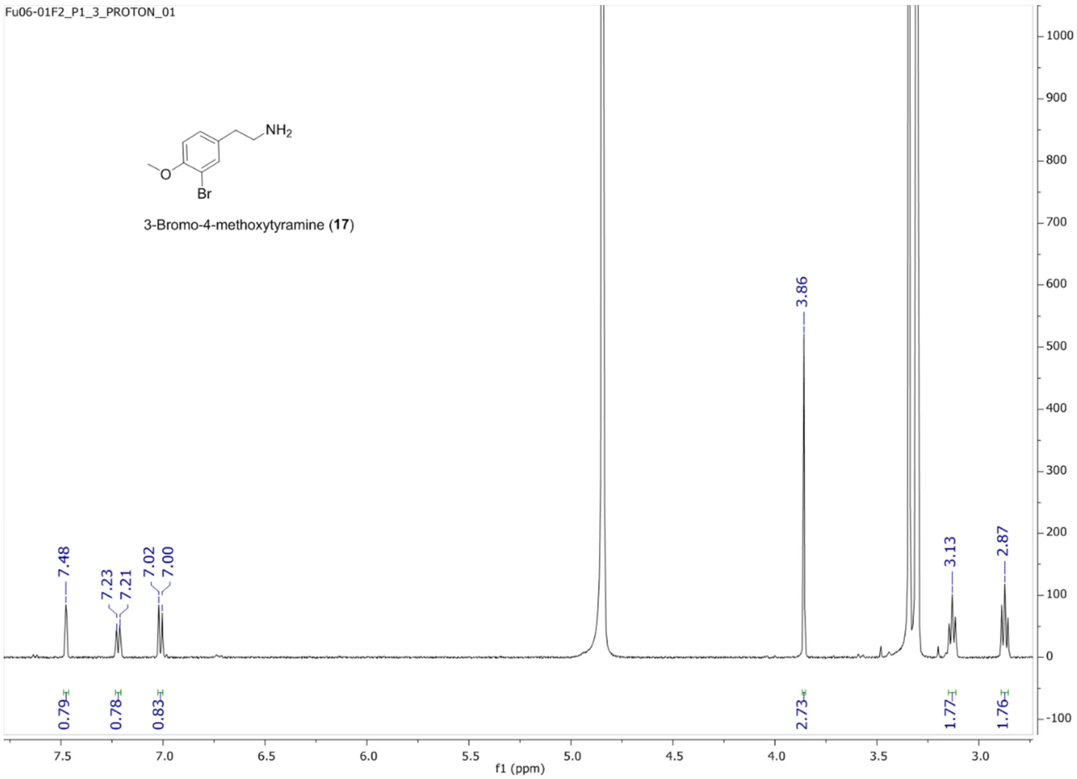

S76. ^{1}H NMR spectrum of **17** (500 MHz, MeOH-d_4).

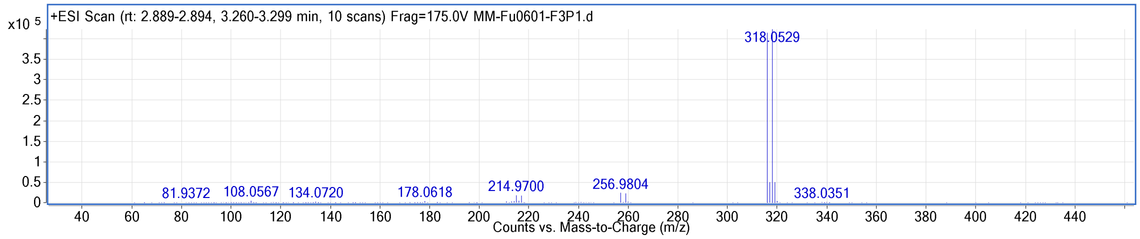

S77. ESI(+)-HRMS analysis of **18**

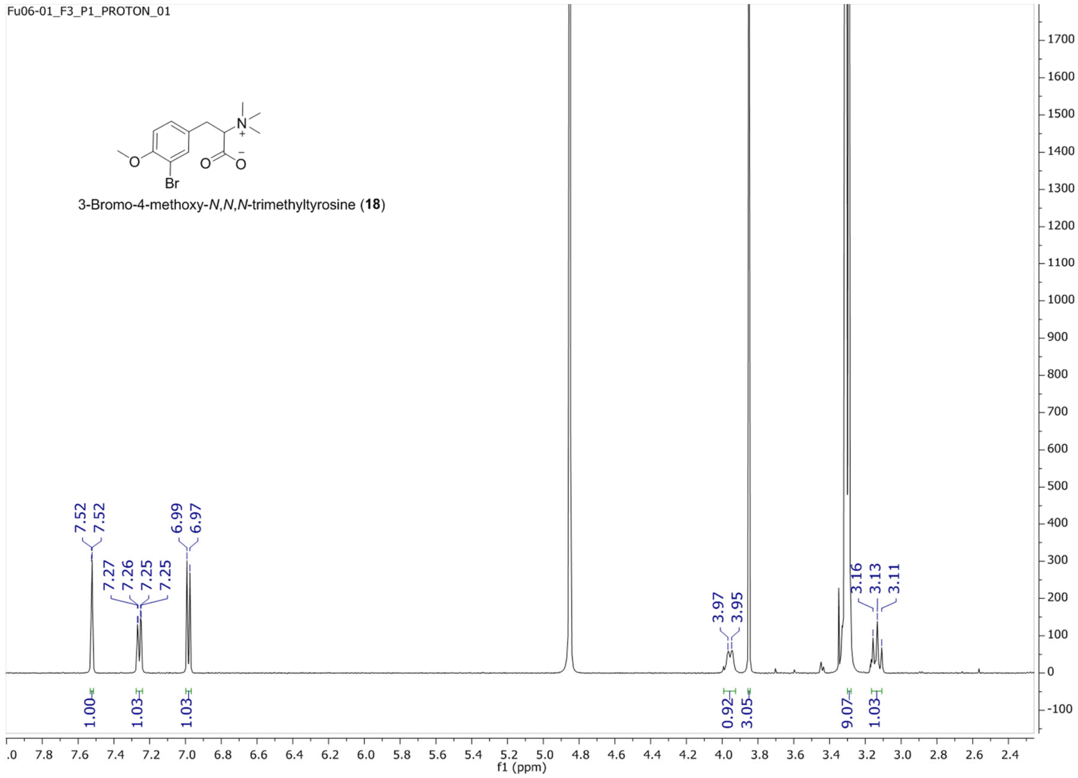

S78. ^{1}H NMR spectrum of **18** (500 MHz, MeOH-d_4).

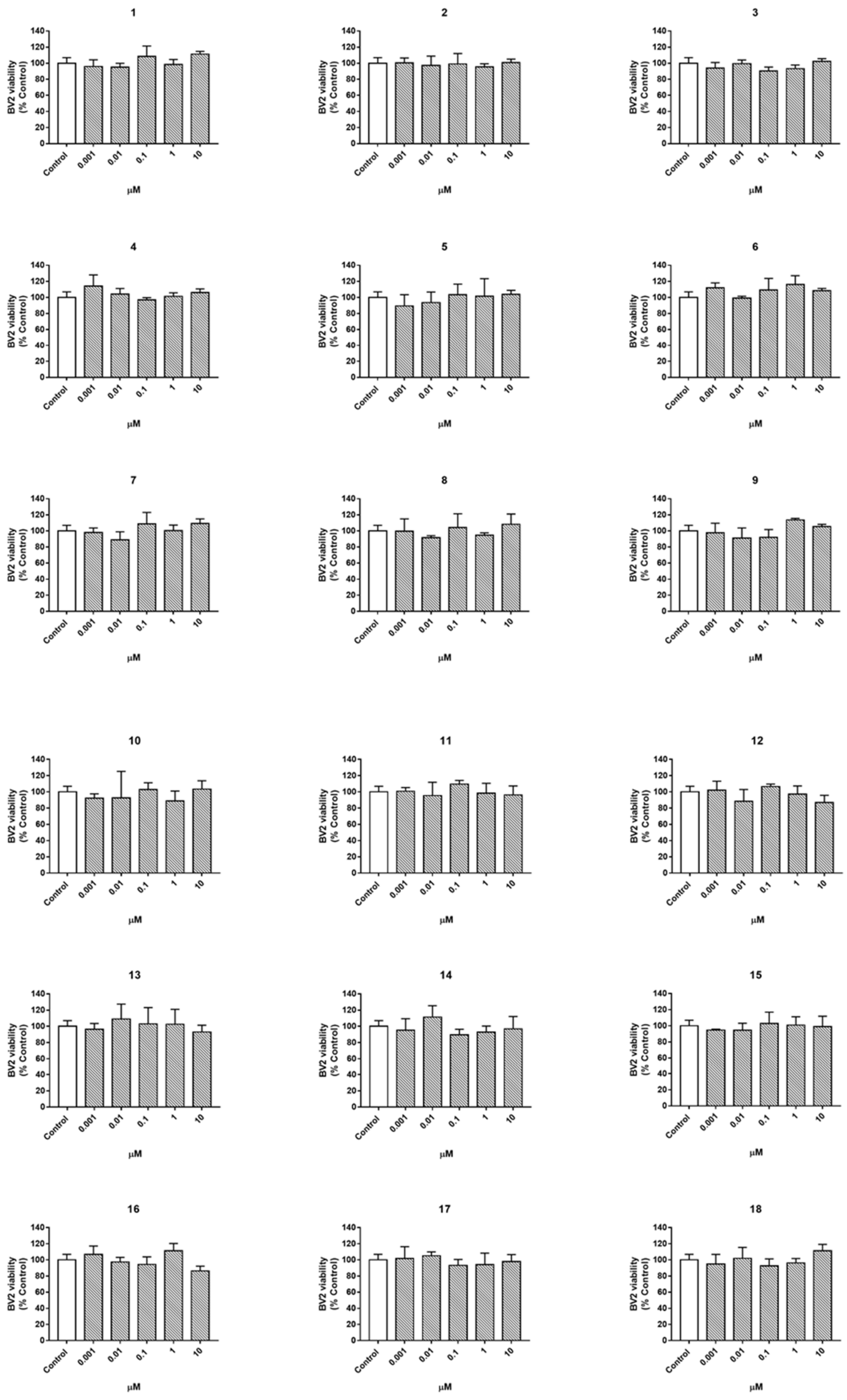

S79. Cell viability of brominated alkaloids over microglia BV2 cell line.
Cells were treated with compounds (0.001, 0.01, 0.1, 1 and 10 µM) for 24 hours. Cell viability was determined using MTT test. Dates are represented in percentage of cells control, being the result of mean absorbance ± SEM of three independent experiments done in triplicate.

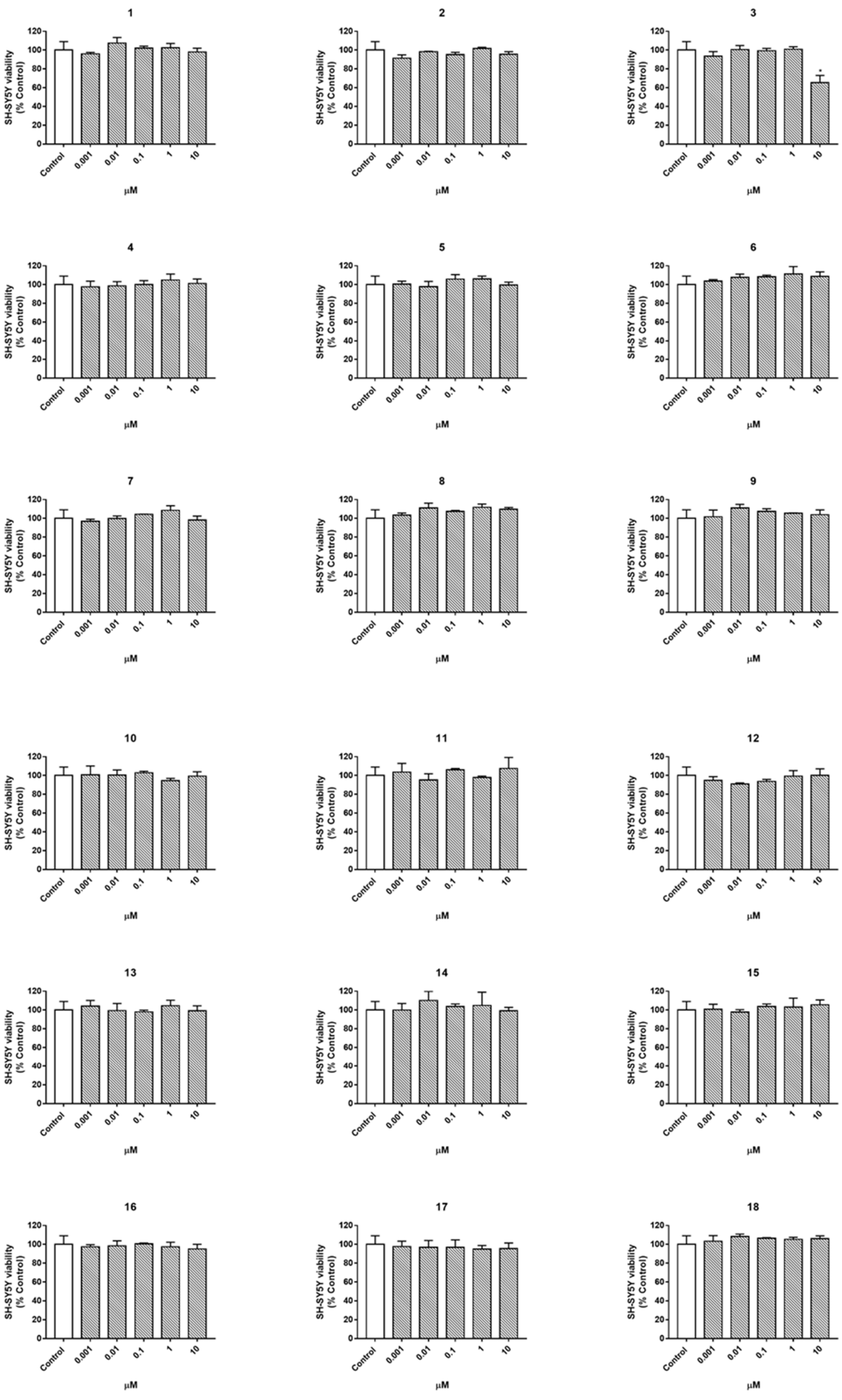

S80. Cell viability of brominated alkaloids over neuroblastoma SH-SY5Y cell line.
Cells were treated with compounds (0.001, 0.01, 0.1, 1 and 10 μM) for 24 hours. Cell viability was determined using MTT test. Dates are represented in percentage of cells control, being the result of mean absorbance ± SEM of three independent experiments done in triplicate.

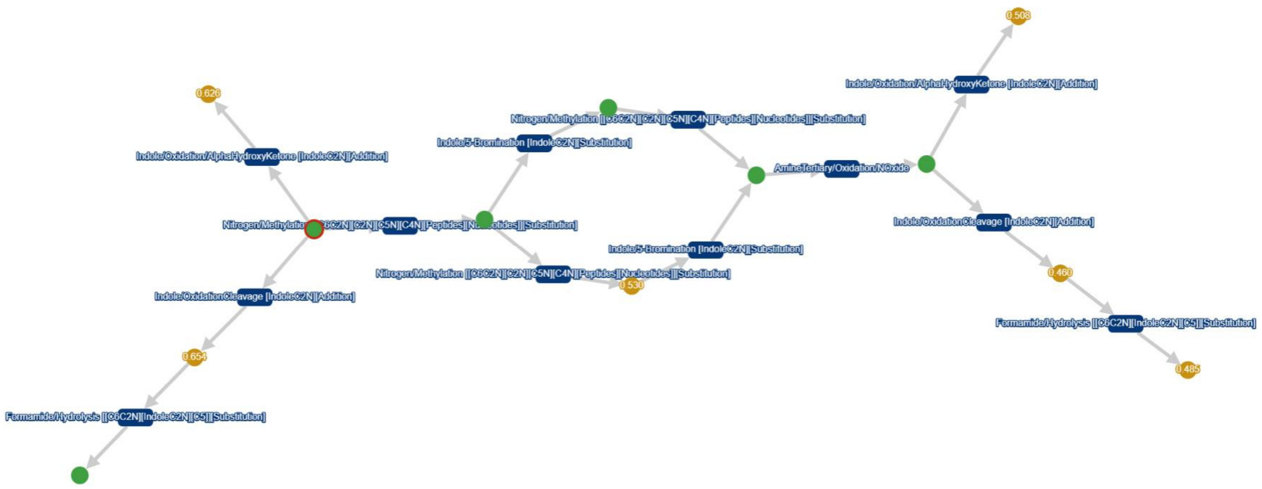

S81. Cosine values of all newly identified compounds in orange in the MetWork software.

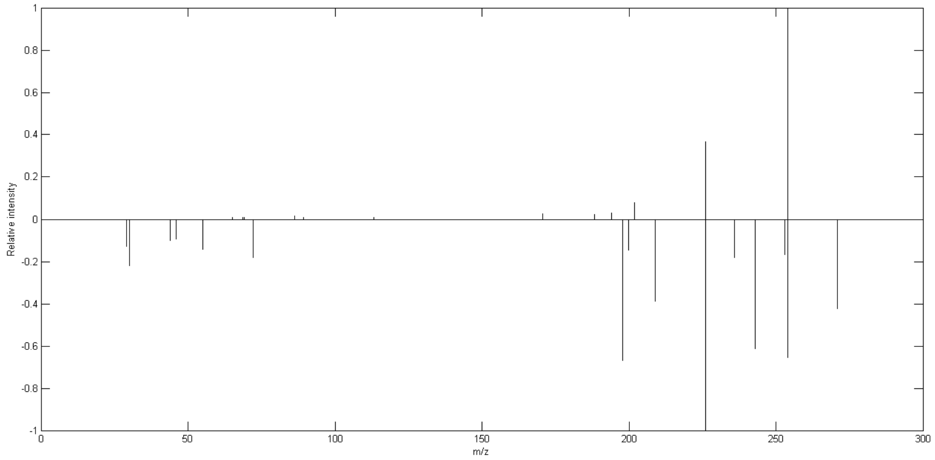

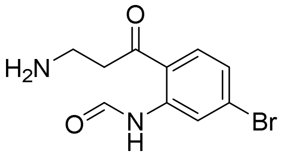

S82. Comparison between the experimental and calculated MS/MS spectrum of the minor compound below.
